# Supplementary material for: Prevention of excitotoxicity‐induced processing of BDNF receptor TrkB‐FL leads to stroke neuroprotection
Source: EMBO Mol Med. 2019 Jun 3;11(7):e9950. doi: 10.15252/emmm.201809950 (PMC6609917; doi:10.15252/emmm.201809950)
Supplement: Supplementary file 1 — Appendix [file EMMM-11-e9950-s001.pdf]

APPENDIX

“Prevention of excitotoxicity-induced processing of BDNF receptor TrkB-FL leads to stroke neuroprotection”

Table of contents

- Appendix Figure S1………………………………………………………….. 2
- Appendix Figure S2………………………………………………………….. 3
- Appendix Figure S3………………………………………………………….. 4
- Appendix Figure S4………………………………………………………….. 5
- Appendix Table S1…………………………………………………………….. 6

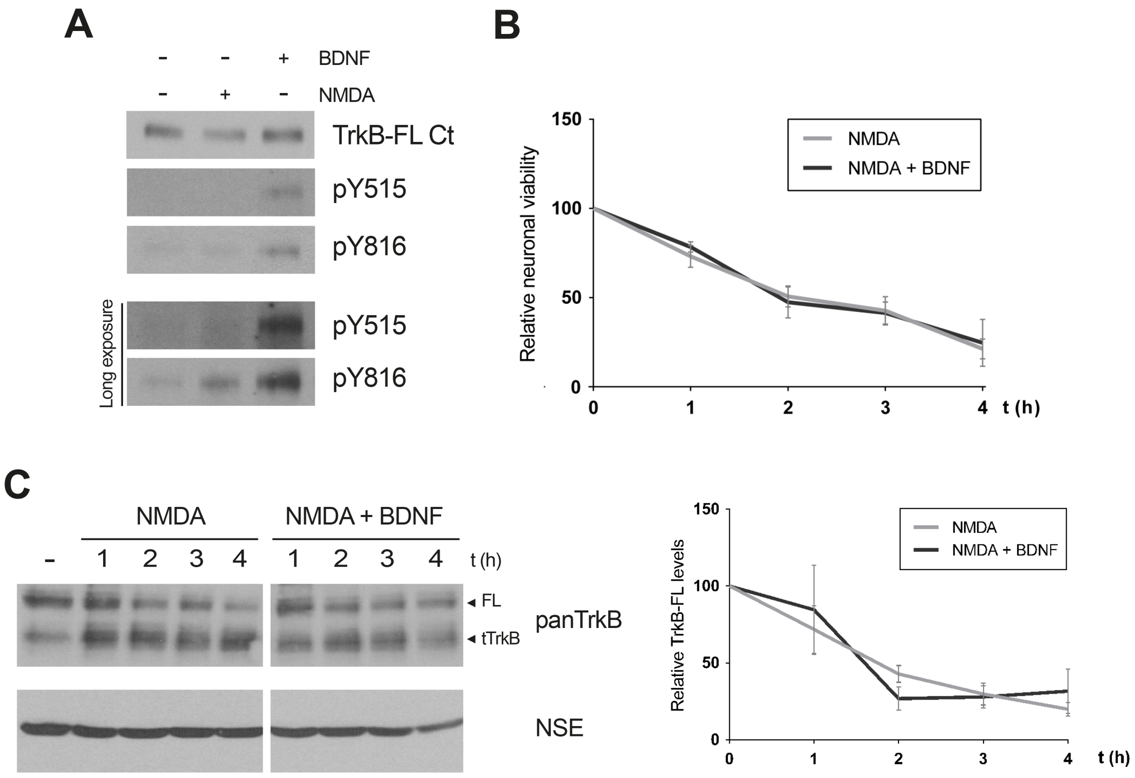

Appendix Figure S1. Analysis of primary neuronal cultures treated with NMDA and BDNF.

(A) Analysis of TrkB-FL phosphorylation. Cultures were treated with NMDA (100 µM) or BDNF (100 ng/ml) for 1 h. Total lysates were analyzed by WB with TrkB-FL Ct or phospho-specific TrkB antibodies for residues Y515 and Y816. Two different exposures are presented to highlight inducibility by BDNF (upper panels) or basal levels of TrkB-FL phosphorylation (lower panels).

(B) Effect of BDNF pretreatment on neuronal death induced by excitotoxicity. Cultures were preincubated 1 h with BDNF or vehicle and treated with NMDA for 1-4 h as before. Values for each time point were represented relative to those of the untreated cultures. Mean ± SEM of three independent experiments is given. Data were analyzed by a two-way ANOVA test followed by *post-hoc* Bonferroni test comparing values for each time point corresponding to NMDA or NMDA + BDNF. No significant differences were found.

(C) Effect of BDNF pretreatment on TrkB levels. Immunoblot analysis of cultures treated as above with NMDA for 1-4 h using panTrkB and NSE antibodies. Normalized TrkB-FL levels are presented relative to values obtained in untreated cells. Means ± SEM are given (n = 5). Results were analyzed as above.

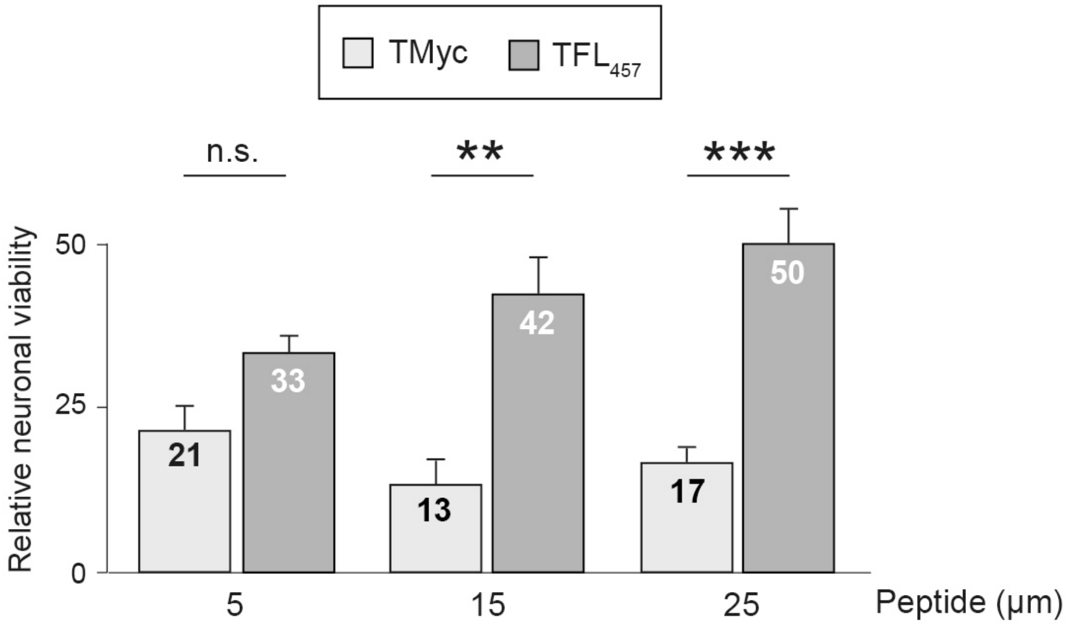

Appendix Figure S2. Dose-dependent effect of TFL_{457} on neuronal viability after induction of excitotoxicity.

Neuronal viability was established in cultures preincubated with TMyc o TFL_{457} (5, 15 o 25 μM) for 30 min and treated with NMDA for 4 h. Average values are represented relative to those obtained in neurons incubated with the same concentration of peptide but no NMDA, arbitrarily given a 100% value (n = 6). Statistical significance was determined by one-way ANOVA followed by Tukey's HSD *post-hoc* test, comparing for each peptide concentration the neuronal viability of cultures preincubated with TMyc or TFL_{457} (n.s. = non-significant, p = 0.38; **p = 0.003; ***p = 0.000002).

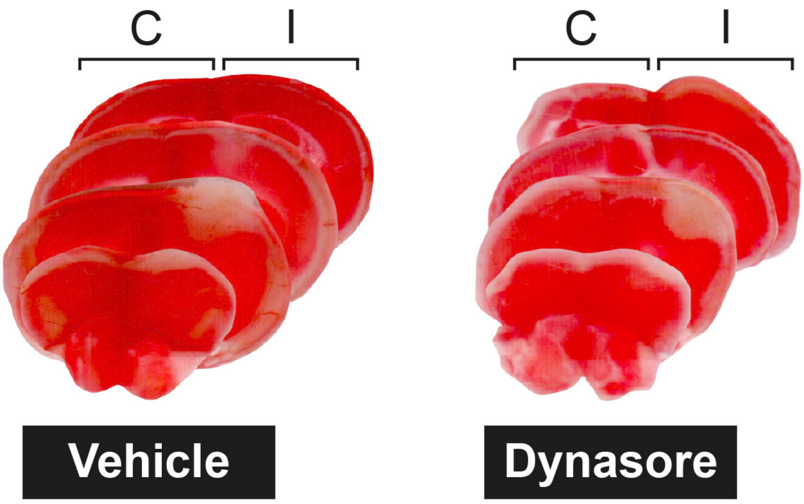

Appendix Figure S3. Assessment of brain ischemic damage at early times after inhibition of dynamin-dependent endocytosis.

Mice were injected with dynasore (30 mg/Kg) or vehicle before damage induction and sacrificed 3 h after brain insult. We present representative 2 mm brain coronal slices, briefly stained with TTC to facilitate dissection of the infarcted area of the ipsilateral hemisphere (I) and the corresponding region of the contralateral area (C). At this early time, emergent cortical infarcts that evolve with time can be observed.

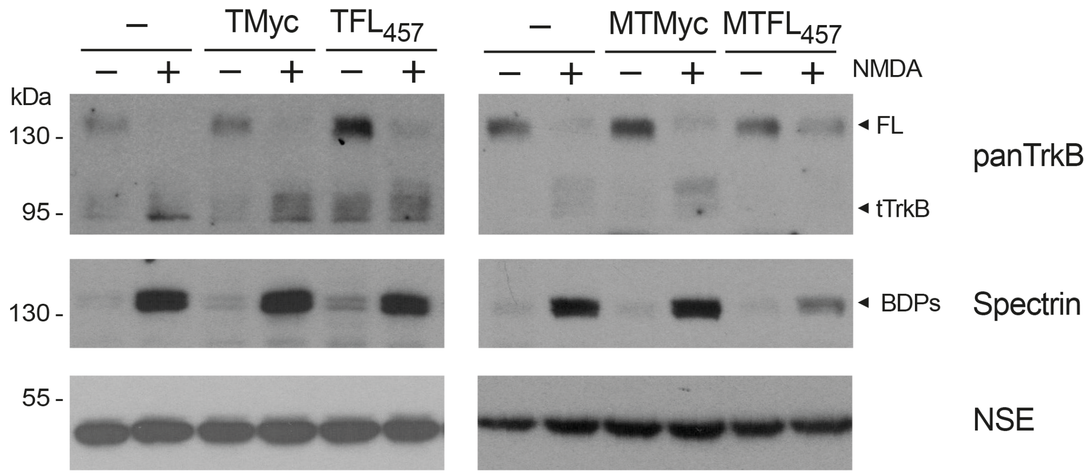

Appendix Figure S4. MTFL$_{457}$ interferes TrkB-FL downregulation induced by *in vitro* excitotoxicity.

Primary cultures were preincubated for 30 min with with 25 μM of MTMyc, MTFL$_{457}$, or no peptide before induction of excitotoxicity by NMDA treatment (2 h). Results were compared to those obtained with the non-modified peptides, TMyc and TFL$_{457}$, having free α-amino and carboxyl groups in their N-ter and C-ter.

Appendix Table S1. Description of reagents or resources used in the study

REAGENT or RESOURCE	SOURCE	IDENTIFIER
Antibodies		
Rabbit polyclonal anti-**CREB** (1:1000)	Cell Signaling	Cat#9197; RRID:AB_331277
Rabbit polyclonal anti-**pS133-CREB** (1:1000)	Millipore	Cat#06-519; RRID:AB_310153
Mouse monoclonal anti-**GluN1** (1:1000)	BD Biosciences	Cat#556308; RRID:AB_396353
Goat polyclonal anti-**GluN2A** (1:250)	Santa Cruz	Cat#sc-1468; RRID:AB_670223
Mouse monoclonal anti-**MEF2D** (1:1000)	BD Biosciences	Cat# 610774, RRID:AB_398095
Mouse monoclonal anti-RBFOX3/**NeuN** (1:100)	Novus	Cat#NBP1-92693SS; RRID:AB_1103747
Rabbit polyclonal anti-neuronal-specific enolase (**NSE**) (1:50000)	Millipore	Cat#AB951; RRID:AB_92390
Mouse monoclonal anti-**phosphotyrosine** (4 µg/mg of protein)	Millipore	Cat#05-321; RRID:AB_309678
Mouse monoclonal anti-**spectrin** alpha chain (1:50000)	Millipore	Cat#MAB1622; RRID:AB_94295
Rabbit polyclonal anti-**TrkB-FL (C-ter region)** (1:500)	Santa Cruz	Cat#sc-11; RRID:AB_632554
Rabbit polyclonal anti-**TrkB-T1** (isoform-specific C-ter) (1:500)	Santa Cruz	Cat#sc-119; RRID:AB_632559
Mouse monoclonal anti-TrkB (extracel. seq. common to all TrkB isoforms; **panTrkB**) (1:500)	Santa Cruz	Cat#sc-136990; RRID:AB_2155262
Rabbit polyclonal anti-pY490-TrkA (anti-**pY515-TrkB**) (1:500)	Cell Signaling	Cat#9141; RRID:AB_2298805
Rabbit monoclonal anti-**pY816-TrkB** (1:500)	Boster	Cat#P01388
Goat anti-mouse IgG Alexa Fluor 546 (1:500)	Molecular Probes	Cat#A-11030; RRID:AB_144695
Donkey anti-goat IgG-HRP (1:5000)	Santa Cruz	Cat#sc-2056; RRID:AB_631730
Goat anti-mouse-HRP (1:5000)	Santa Cruz	Cat#sc-2005; RRID:AB_631736
Goat anti-rabbit-HRP (1:5000)	Santa Cruz	Cat#sc-2004; RRID:AB_631746
Donkey anti-rabbit IgG-heavy and light chain-HRP (1:50000)	Bethyl	Cat#A120-108P; RRID:AB_10892625
Donkey anti-mouse IgG-heavy and light chain-HRP (1:50000)	Bethyl	Cat#A90-137P; RRID:AB_1211460
Chemicals, Peptides, and Recombinant Proteins		
**Ara C** (used at 10 µM)	Sigma-Aldrich	Cat#C1768; CAS: 147-94-4
Recombinant Human/Murine/Rat **BDNF** (used at 100 ng/ml)	PeproTech	Cat#450-02
EZ-Link Sulfo-NHS-SS-**Biotin** (used at 0.5 mg/ml)	Thermo Fisher	Cat#21331
Calpain inhibitor III (**CiIII**) (used at 10 µM)	Calbiochem	Cat#208722; CAS: 88191-84-8
**Calpeptin** (used at 10 µM)	Calbiochem	Cat#03-34-0051; CAS: 117591-20-5
**DAPI** (used at 0.5 or 5 µg/ml)	Molecular Probes	Cat#D1306
**DL-AP5** (used at 200 µM)	Tocris Bioscience	Cat#0101; CAS: 76326-31-3
**Dynasore** hydrate (used at 30 mg/kg)	Sigma-Aldrich	Cat#D7693; CAS: 1202867-00-2

**Fluorescein Avidin** D	Vector Laboratories	Cat#A 2001
**Fluoro-Jade C** (used at 1 µg/ml)	Millipore	Cat#AG325
**Glycine** (used at 10 µM)	Bio-Rad	Cat#161-0718
**GM6001** (used at 10 µM)	Tocris	Cat#2983;<br>CAS: 142880-36-2
**KG-501** (used at 10 µM)	Sigma-Aldrich	Cat#70485;<br>CAS: 18228-17-6
**Laminin** (used at 4 µg/ml)	Sigma-Aldrich	Cat#L2020;<br>CAS: 114956-81-9
**MTT** (used at 0.5 mg/ml)	Sigma-Aldrich	Cat#M5655;<br>CAS: 298-93-1
**NMDA** (used at 20 or 100 µM as indicated)	Tocris	Cat#0114;<br>CAS: 6384-92-5
**PhosSTOP** phosphatases inhibitor cocktail tablets	Roche	Cat#04 906 837 001
**Poly-L-Lysine** (used at 100 µg/ml)	Sigma-Aldrich	Cat#P1524;<br>CAS: 25988-63-0
**Prolong** Diamond antifade reagent	Molecular Probes	Cat#P36970
Complete **protease inhibitor** cocktail tablets	Roche	Cat#11 697 498 001
Recombinant **Protein G Agarose**	Thermo Fisher	Cat#15920010
**Rose Bengal** (used at 20 mg/kg)	Sigma-Aldrich	Cat#R3877;<br>CAS: 632-69-9
**Streptavidin** resin	GenScript	Cat#L00353
**TTC** (used at 2%)	Sigma-Aldrich	Cat#T8877;<br>CAS: 298-96-4
**U0126** (used at 300 nM)	Cell Signaling	Cat#9903;<br>CAS: 109511-58-2
**U-73122** (used at 5 µM)	Sigma-Aldrich	Cat#U6756;<br>CAS: 112648-68-7
**Wortmannin** (used at 100 nM)	Ascent Scientific	Cat#Asc-148;<br>CAS: 19545-26-7
Cell-penetrating peptides		
**TMyc** (*YGRKKRRQRRR*AEEQKLISEEDLLR)	GenScript	N/A
**MTMyc** (Ac-*YGRKKRRQRRR*AEEQKLISEEDLLR-NH_2)	GenScript	N/A
**Bio-TMyc** (Biotin-Ahx-*YGRKKRRQRRR*AEEQKLISEEDLLR-NH_2)	GenScript	N/A
**TFL_{457}** (*YGRKKRRQRRR*HSKFGMKGPASVIS)	GenScript	N/A
**$MTFL_{457}$** (Ac-*YGRKKRRQRRR*HSKFGMKGPASVIS-NH_2)	GenScript	N/A
**Bio-TFL_{457}** (Biotin-Ahx-*YGRKKRRQRRR*HSKFGMKGPASVIS-NH_2)	GenScript	N/A
**TFL_{482}** (*YGRKKRRQRRR*HISNGSNTPSSSEG)	GenScript	N/A
**TFL_{518}** (*YGRKKRRQRRR*ITNSQLKPDTFVQH)	GenScript	N/A
**TFL_{541}** (*YGRKKRRQRRR*ELGEGAFGKVFLAE)	GenScript	N/A
**Bio-NA-1** (Biotin-Ahx-*YGRKKRRQRRR*KLSSIESDV-NH_2)	GenScript	N/A
Primer sequences for real-time PCR		
GluN1-F (all isoforms): TCCACCAAGAGCCCTTCGTG	Sigma-Aldrich	N/A
GluN1-R (all isoforms): GCCCCGTACAGATCACCTTC	Sigma-Aldrich	N/A
GluN2A-F: ACGACTGGGACTACAGCCTG	Sigma-Aldrich	N/A
GluN2A-R: CTTCTCTGCCTGCCCATAGC	Sigma-Aldrich	N/A
TrkB-FL-F (isoform specific): TATCTTCACCCACCTCAAAC	Sigma-Aldrich	N/A
TrkB-FL-R (isoform specific): GAGAGACTTGACCTGAGCAC	Sigma-Aldrich	N/A
TrkB-T1-F (isoform specific): GGGGCTGTGCTGCTTGGT	Sigma-Aldrich	N/A

TrkB-T1-R (isoform specific): GCTGCGGACATCTTTGGAGA	Sigma-Aldrich	N/A
BDNF-F (all isoforms): TGGCTGACACTTTTGAGCAC	Sigma-Aldrich	N/A
BDNF-R (all isoforms): GTTTGCGGCATCCAGGTAAT	Sigma-Aldrich	N/A
GAPDH-F: TTGCCATCAACGACCCCTTC	Sigma-Aldrich	N/A
GAPDH-R: GCCTTGACTGTGCCGTTGAA	Sigma-Aldrich	N/A
NSE-F: ACAGAATGGGGCTGTGTACC	Sigma-Aldrich	N/A
NSE-R: TGGCAACTGTGGGACATGGC	Sigma-Aldrich	N/A
Critical Commercial Assays		
BCA Protein Assay Kit	Thermo Fisher	Cat# 23225
Clarity Western ECL Blotting Substrate	BioRad	Cat# 1705060
Lipofectamine 2000	Life Technologies	Cat#11668019
Experimental Models: Organisms/Strains		
Balb/c inbred mice (Balb/cOlaHsd)	Harlan Laboratories	N/A
Wistar Rat embryos (E18)	In site facility	N/A
Recombinant DNA		
**pIIIBDNF** (170 bp of rat *Bdnf* promoter III, containing a CRE-like element subcloned into pGL3-basic)	(Tao et al., 1998)	N/A
**pCRE** (25-mer oligonucleotide with the sequence of two TrkB CREs subcloned into pTK-Luc)	(Deogracias et al.,2004)	N/A
**pGluN1** (pNRL 5.4 kb; 5.4 kb of rat *Grin1* containing three CREs and one MEF2 subcloned into pGL3-basic)	(Bai et al., 2003)	N/A
**pGluN2A** (-1253 to -210 of mice *Grin2a* promoter with a CRE-like element subcloned into pGL3-basic)	(Desai et al., 2002)	N/A
**pMEF2** (pRSRF; -307 to -242 of *Nur77* promoter with two MEF2 sites subcloned into pGL2-basic)	(Woronicz et al., 1995)	N/A
**pMEF2mut** (two inactivating point mutations in each MEF2 site of pMEF2)	(Woronicz et al., 1995)	N/A
**pTrkB** (-1600trkB-P2-Luc; -1606 to -325 of *Ntrk2* promoter subcloned into pGL3-basic)	(Deogracias et al.,2004)	N/A
Software and Algorithms		
Statistical Package for Social Science (SPSS v.24)	IBM (https://www.ibm.com)	RRID:SCR_002865
CaMPDB (Calpain cleavage prediction using multiple kernel learning; prediction models: MKL, PSSM, SVM linear kernel and SVM RBF kernel)	(DuVerle et al., 2011); http://calpain.org/ predict.rb?cls=substrate	RRID:SCR_011976
GPS-Calpain Cleavage Detector	(Liu et al., 2011); http://ccd.biocuckoo.org/	RRID:SCR_000202
SitePrediction (predict cleavage site of proteases)	(Verspurten et al., 2009); http://www.dmbr.ugent. be/prx/bioit2-public/ SitePrediction/	N/A
Predictions for Entire Proteomes (including SNAP prediction of the effects of single amino acid substitutions on protein function)	(Hecht et al., 2015) (http://www.predictprotein .org/)	RRID:SCR_002803
GeneSilico Metadisorder service	(Kozlowski and Bujnicki, 2012); http://genesilico.pl/ metadisorder/	N/A
ImageJ	https://imagej.net/	RRID:SCR_003070
Other		
Protran Western blotting nitrocellulose membrane	GE Healthcare	Cat#GE10600002; CAS: 9004-70-0
