# Supplementary material for: Prevention of excitotoxicity‐induced processing of BDNF receptor TrkB‐FL leads to stroke neuroprotection
Source: EMBO Mol Med. 2019 Jun 3;11(7):e9950. doi: 10.15252/emmm.201809950 (PMC6609917; doi:10.15252/emmm.201809950)
Supplement: Supplementary file 2 — Expanded View Figures PDF [file EMMM-11-e9950-s002.pdf]

Expanded View Figures

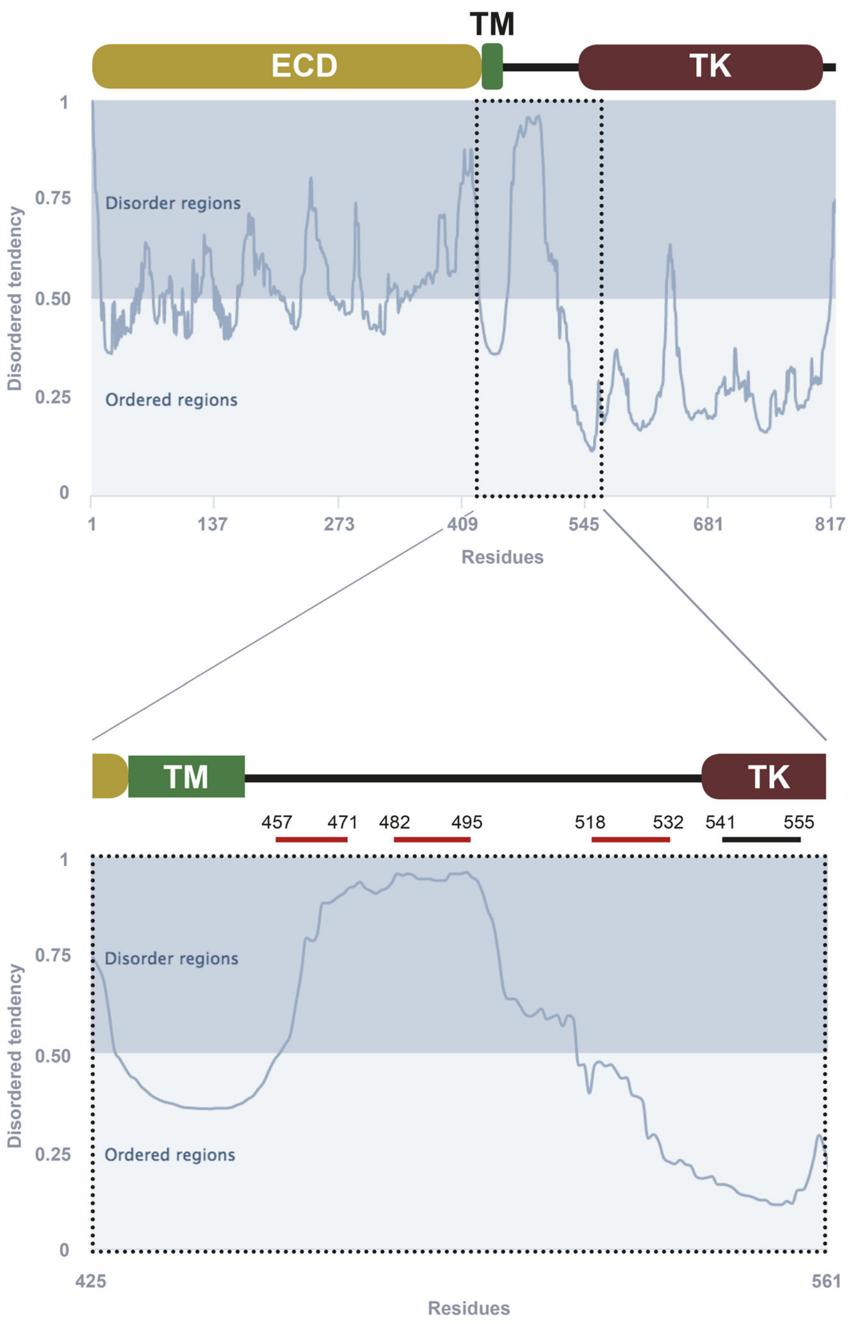

Figure EV1. Analysis of rat TrkB-FL for presence of intrinsically disordered regions (IDRs).

Scheme of TrkB-FL main domains with IDR predictions using the online GeneSilico Metadisorder service. For simplicity, only one plot of the four metapredictions provided is represented (Metadisordermd2). Residues whose disorder probability is over 0.5 are considered as disordered. The juxtamembrane intracellular region is shown with higher detail (lower panel), indicating sequences selected for inclusion in designed CPPs. ECD, extracellular domain; TM, transmembrane region; TK, tyrosine kinase domain.

© 2019 The Authors

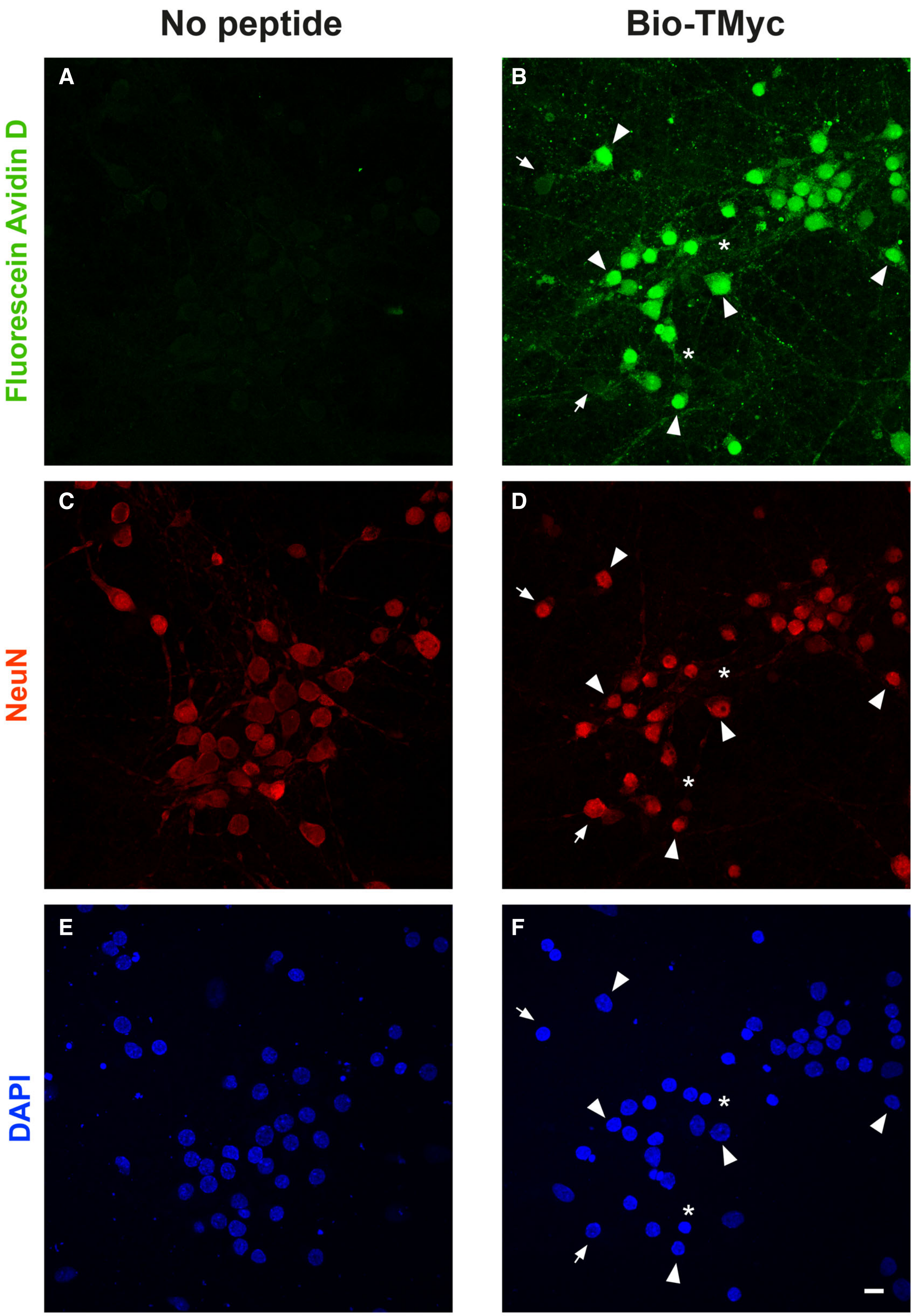

Figure EV2. Quantitation of peptide entry into neurons.

A–F Cultures were treated with no peptide or Bio-TMyc (25 μM, 1 h), detected by Fluorescein Avidin D (green), and stained with anti-NeuN (red) and DAPI (blue). Representative confocal images corresponding to maximum intensity projections were used to quantitate peptide entry into neurons (arrowheads; *n* = 5). A minority of neurons did not incorporate detectable peptide amounts (arrows). Scale bar, 10 μm.

 © 2019 The Authors

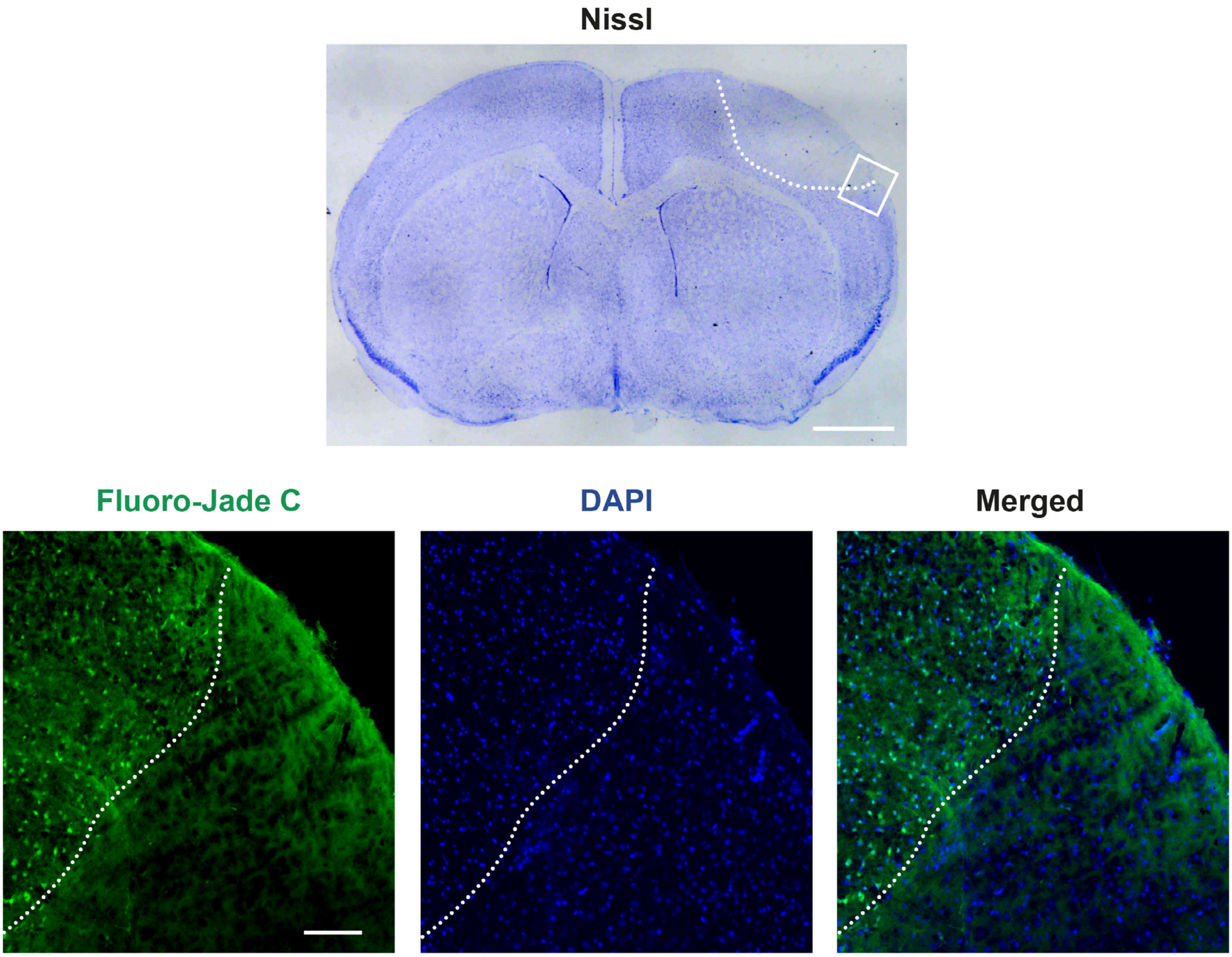

Figure EV3. Demonstration of neuronal degeneration in the cerebral cortex of mice subjected to permanent ischemia by photothrombosis.

Nissl staining of coronal cryosections (30 μm) corresponding to an animal sacrificed 5 h after ischemic induction reveal a hypochromatic area indicative of neuronal injury in the ipsilateral neocortex (highlighted by a white discontinuous line), compared with equivalent regions of the contralateral hemisphere. Adjacent cryosections were analyzed by Fluoro-Jade C (green), which specifically stains degenerating neurons. Cell nuclei were stained with DAPI (blue). The interface between the infarcted and non-infarcted cortical tissue is indicated as before. Scale bar, 1 mm (Nissl); 100 μm (Fluoro-Jade C).

© 2019 The Authors

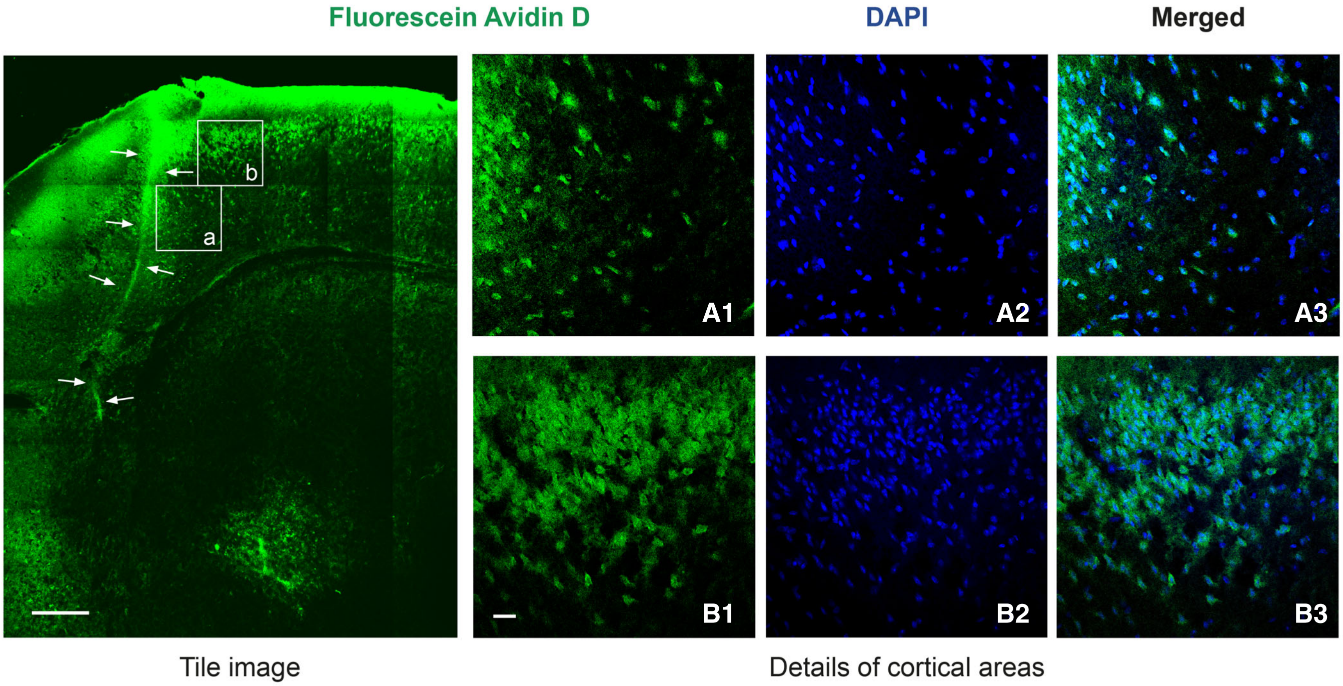

Figure EV4. Detailed analysis of Bio-NA-1 delivery to mice cortex.

Biotinylated NA-1 (Bio-NA-1, 4 nmol/g, i.v.) was detected in coronal sections stained with DAPI. Representative confocal microscopy images correspond to single sections. A tile image of cortical and sub-cortical areas is shown, arrows denoting a heavily fluorescent vessel. Details of two cortical regions in the proximity (a.1–a.3) or further (b.1–b.3) of this vessel are also presented. Scale bar, 250 μm (tile image); 100 μm (details).

 © 2019 The Authors
