# Supplementary material for: Prevention of excitotoxicity‐induced processing of BDNF receptor TrkB‐FL leads to stroke neuroprotection
Source: EMBO Mol Med. 2019 Jun 3;11(7):e9950. doi: 10.15252/emmm.201809950 (PMC6609917; doi:10.15252/emmm.201809950)

**Source data Appendix Figure S1**

**Panel A**

TrkB-FL Ct


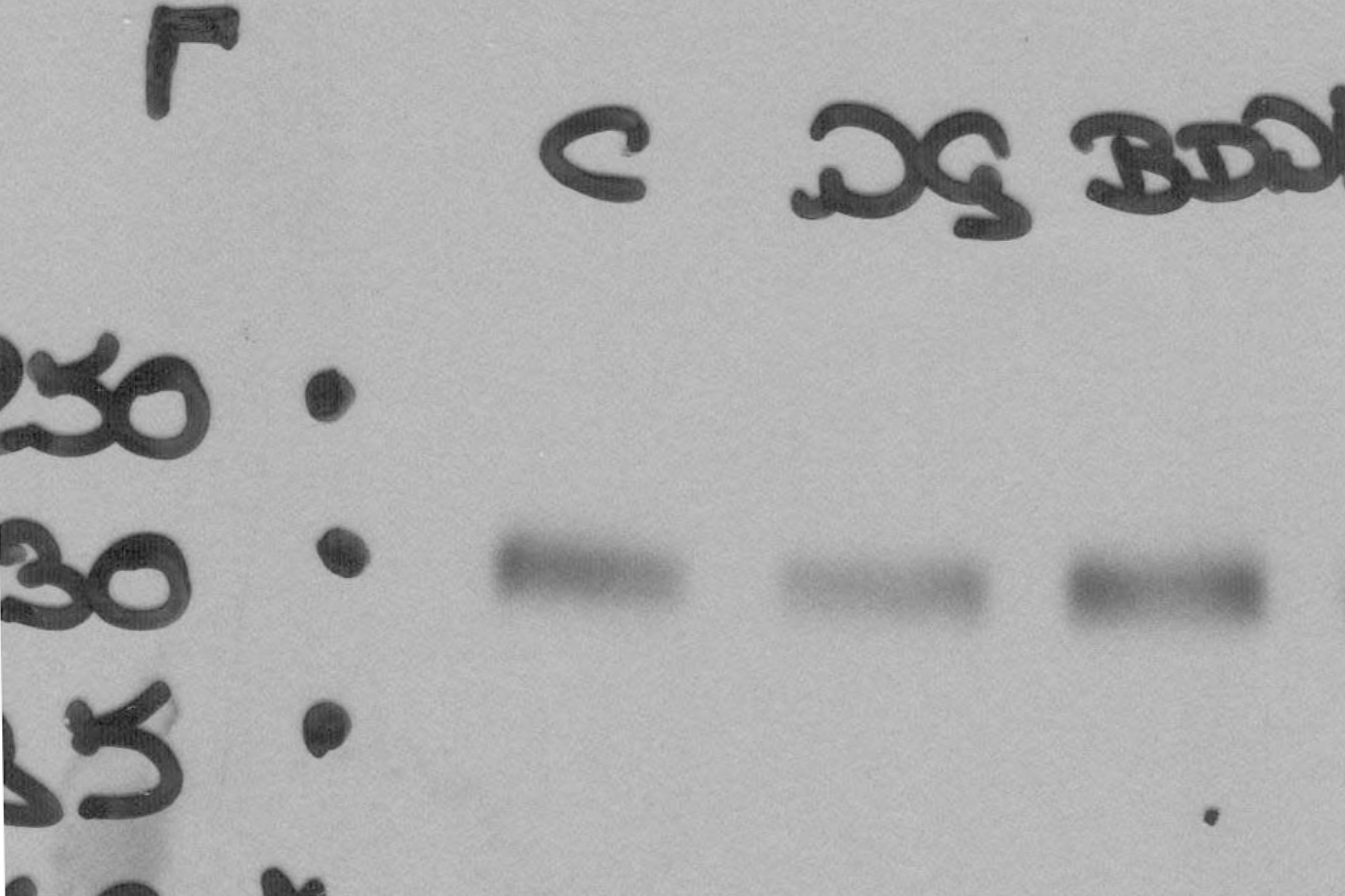


Anti-pY515


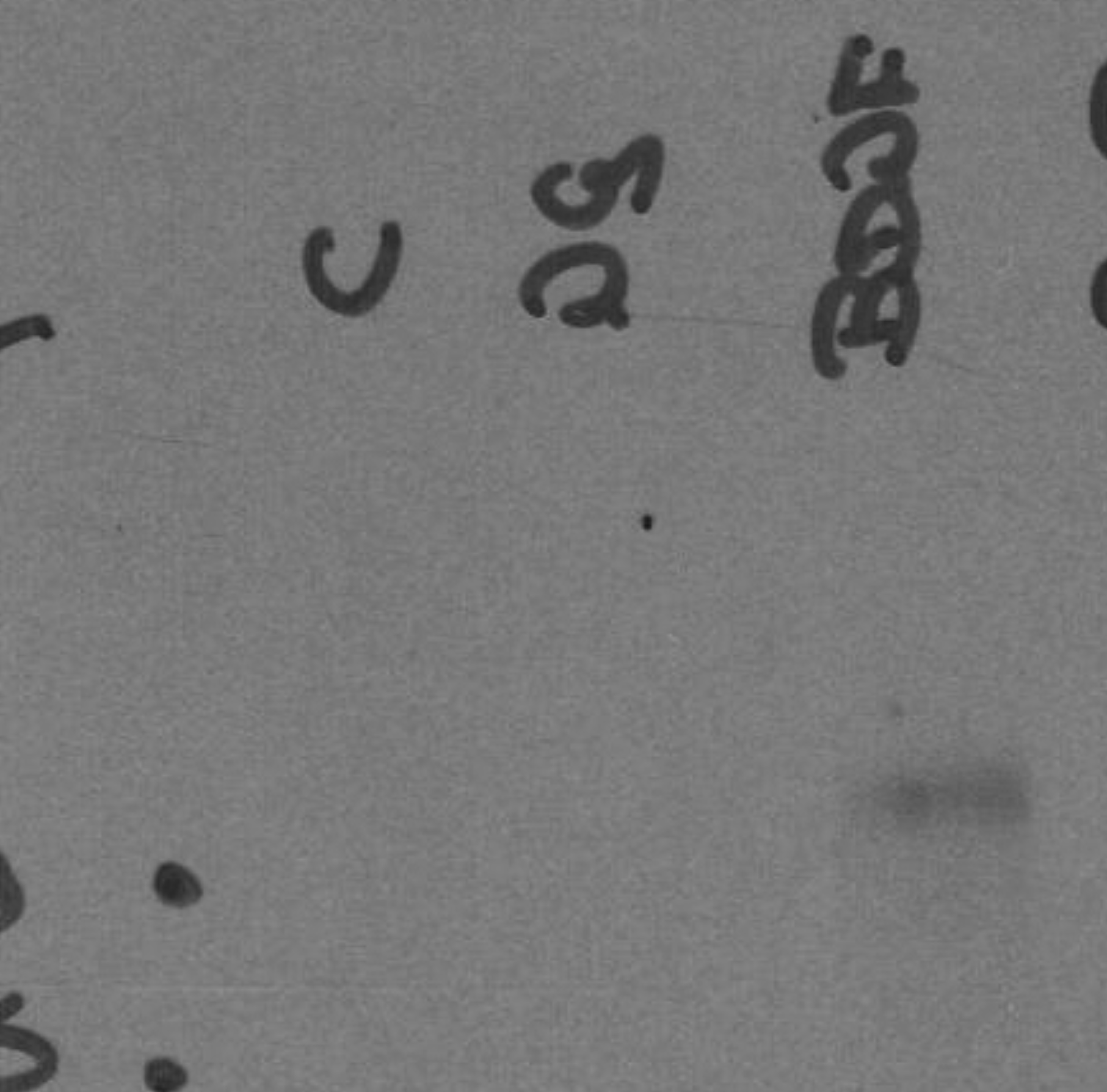


Anti-pY816


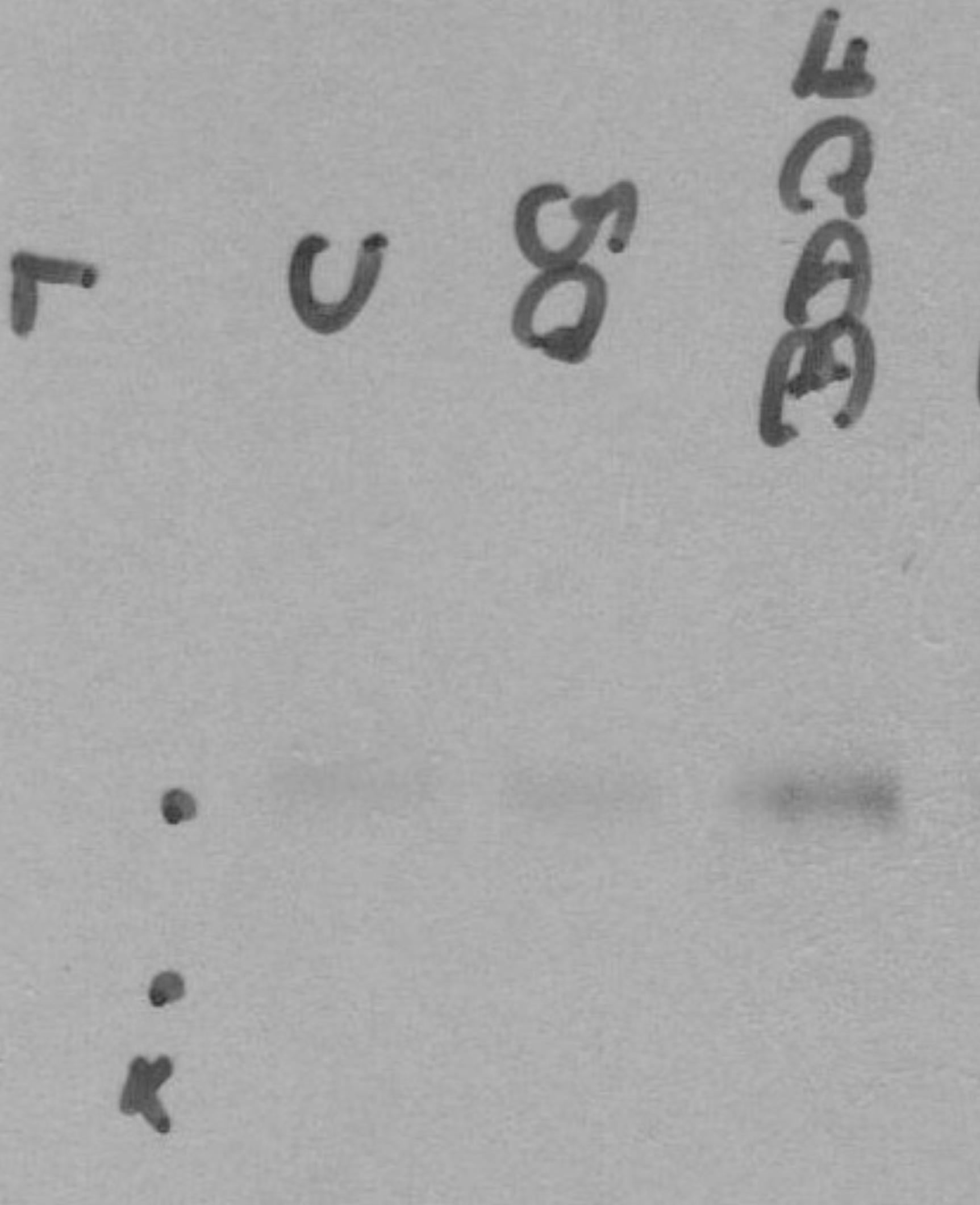


Anti-pY515 (long exposure)


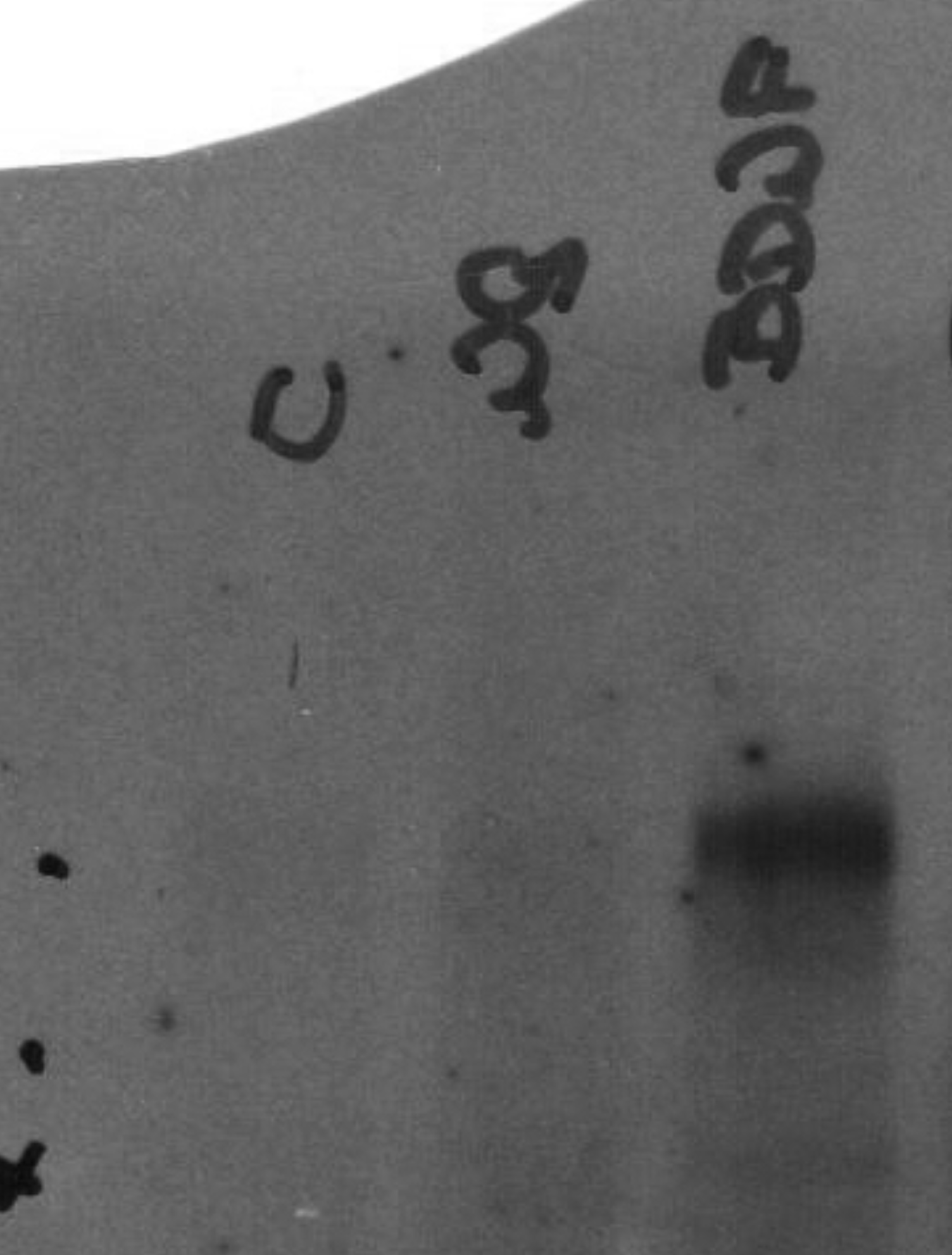


Anti-pY816 (long exposure)


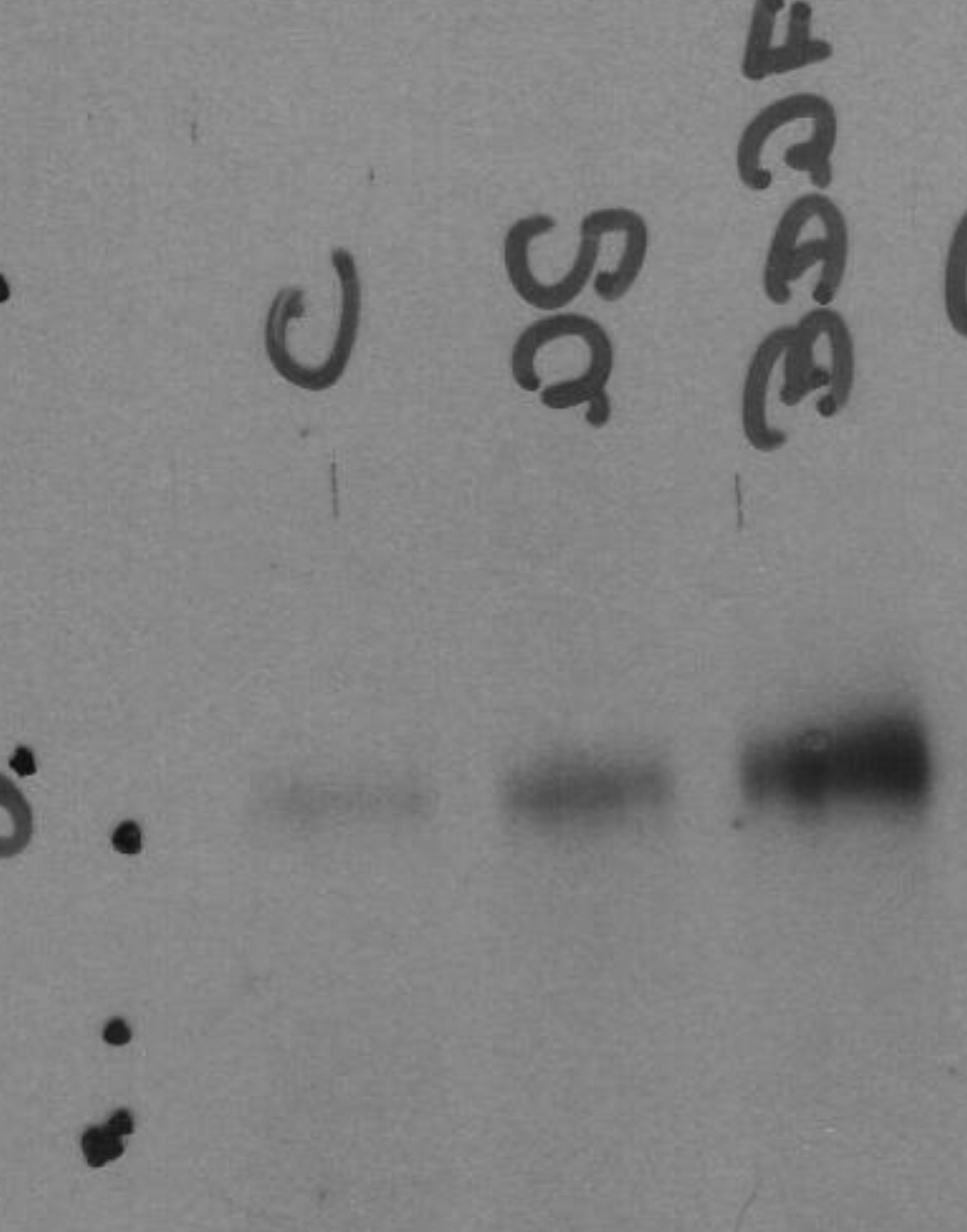


**Panel C**

panTrkB


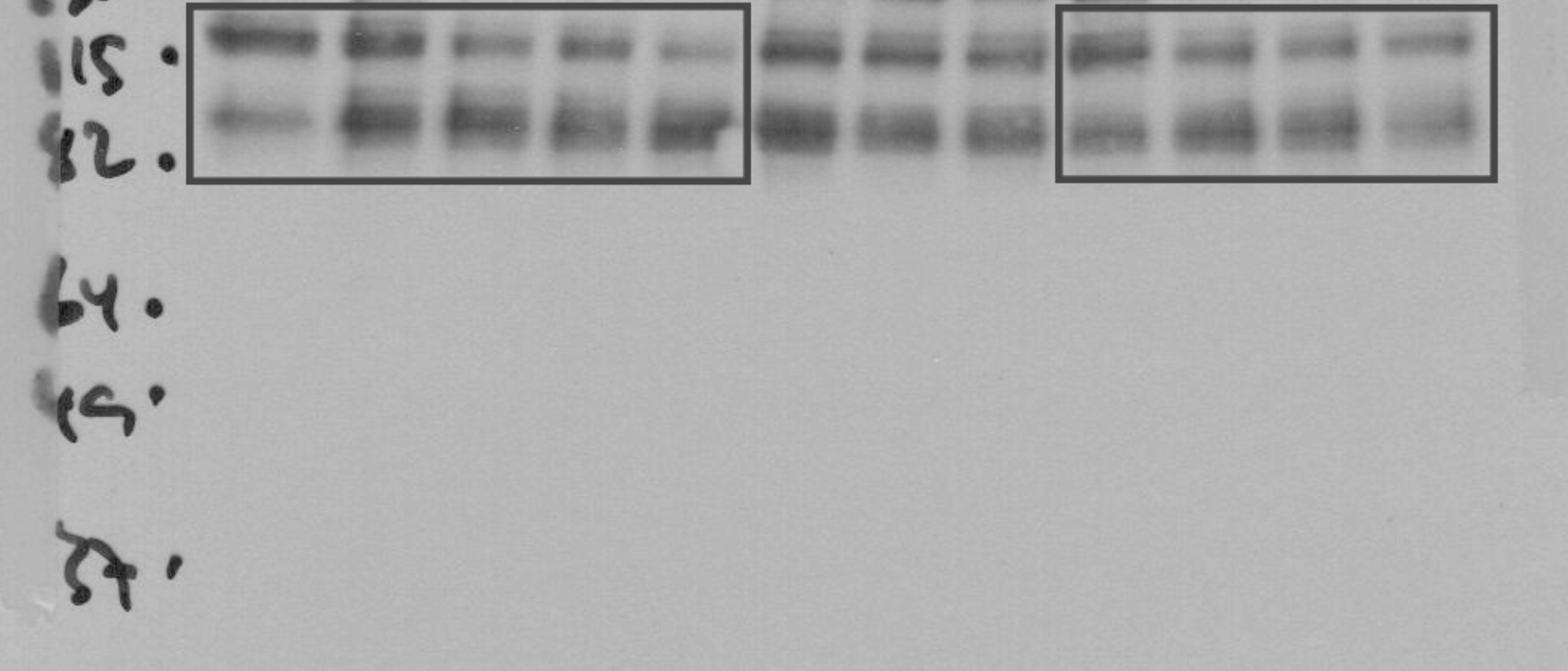


NSE


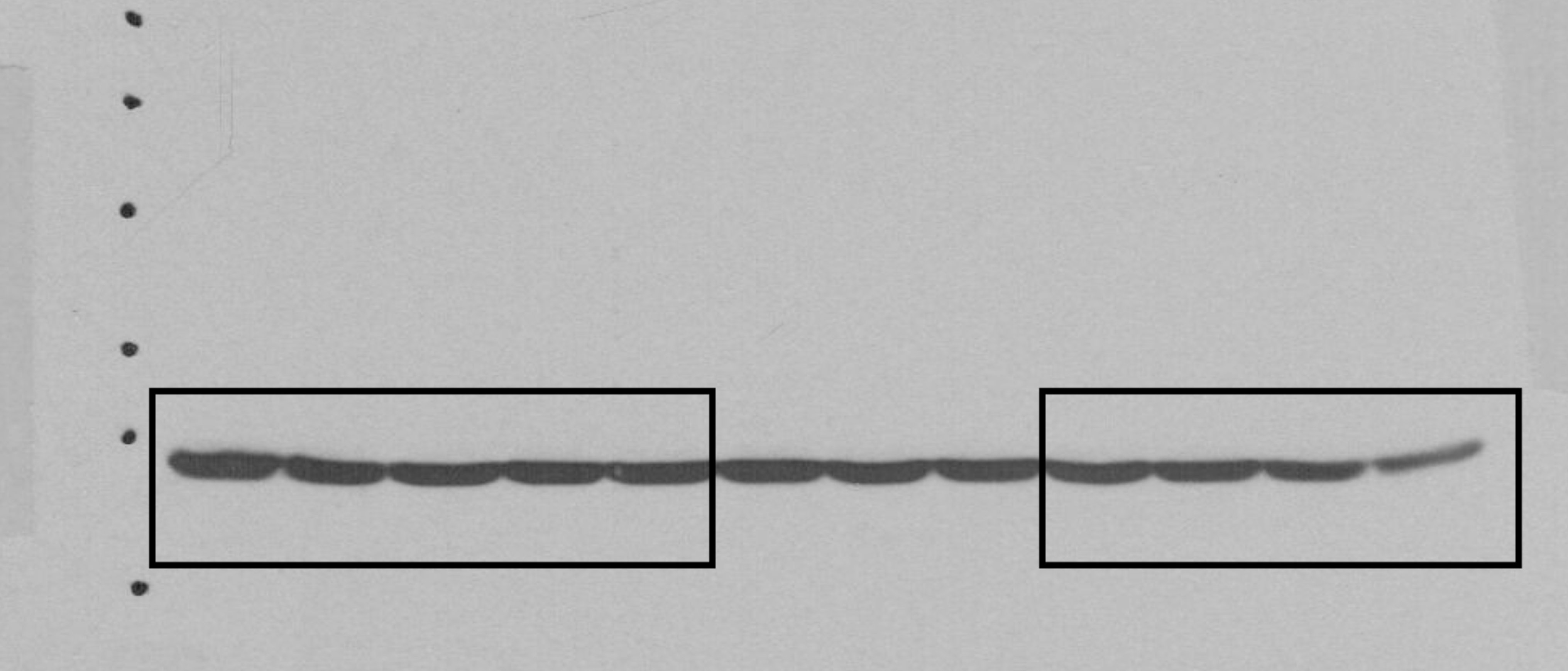

Supplement: Supplementary file 3 — Source Data for Appendix [file EMMM-11-e9950-s008.zip › appendix/Source_data_Appendix_Figure_S1.docx]
