# Supplementary material for: Prevention of excitotoxicity‐induced processing of BDNF receptor TrkB‐FL leads to stroke neuroprotection
Source: EMBO Mol Med. 2019 Jun 3;11(7):e9950. doi: 10.15252/emmm.201809950 (PMC6609917; doi:10.15252/emmm.201809950)

**Source data Figure 2**

**Figure 2A**

Anti-Pan TrkB


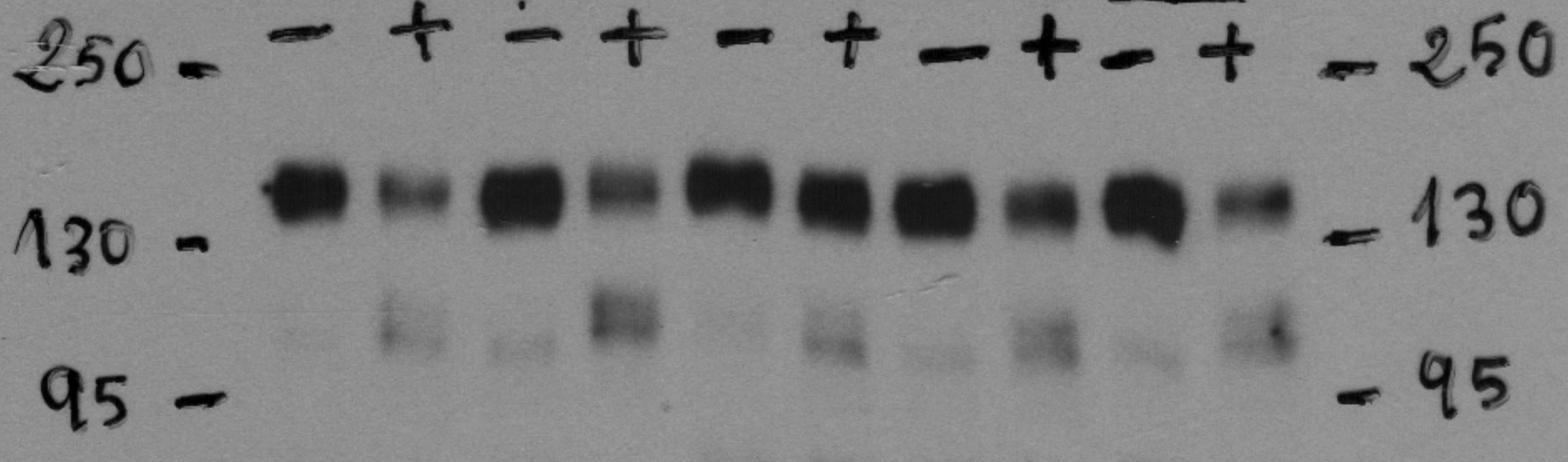


Anti-Spectrin


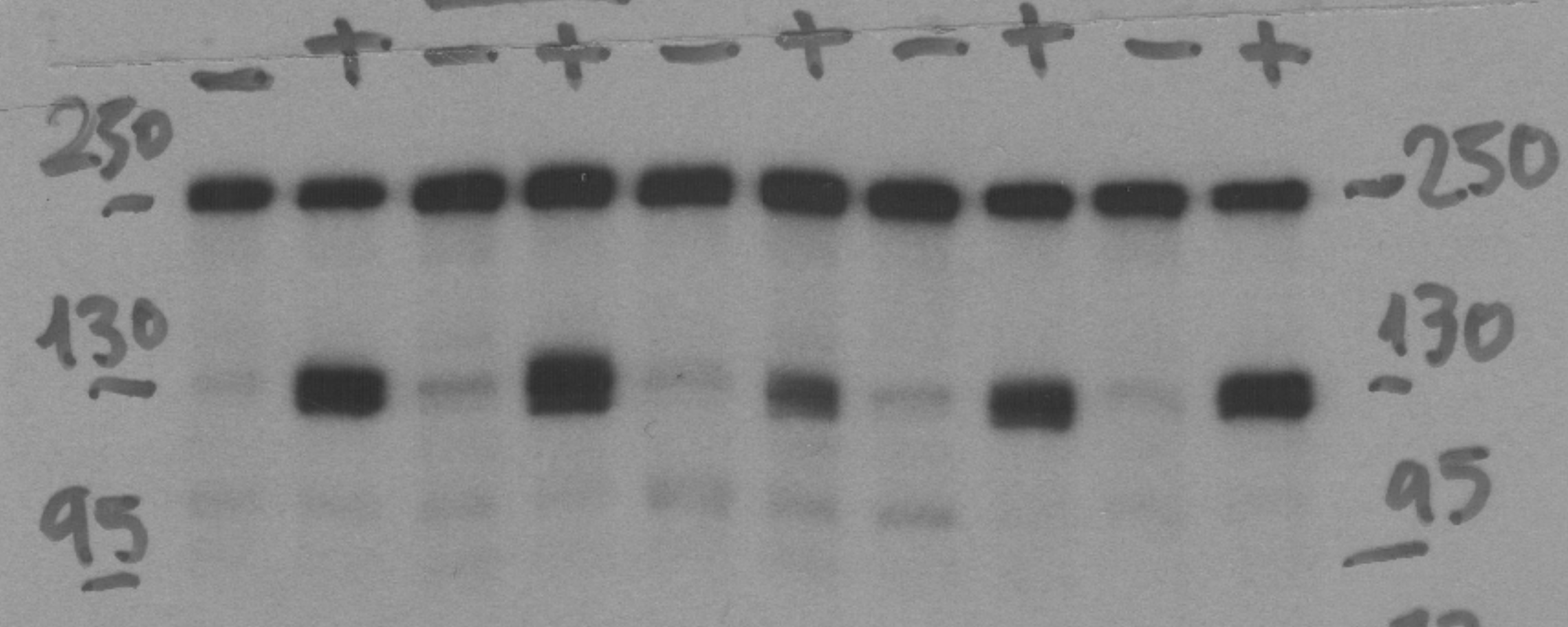


Anti-NSE∫


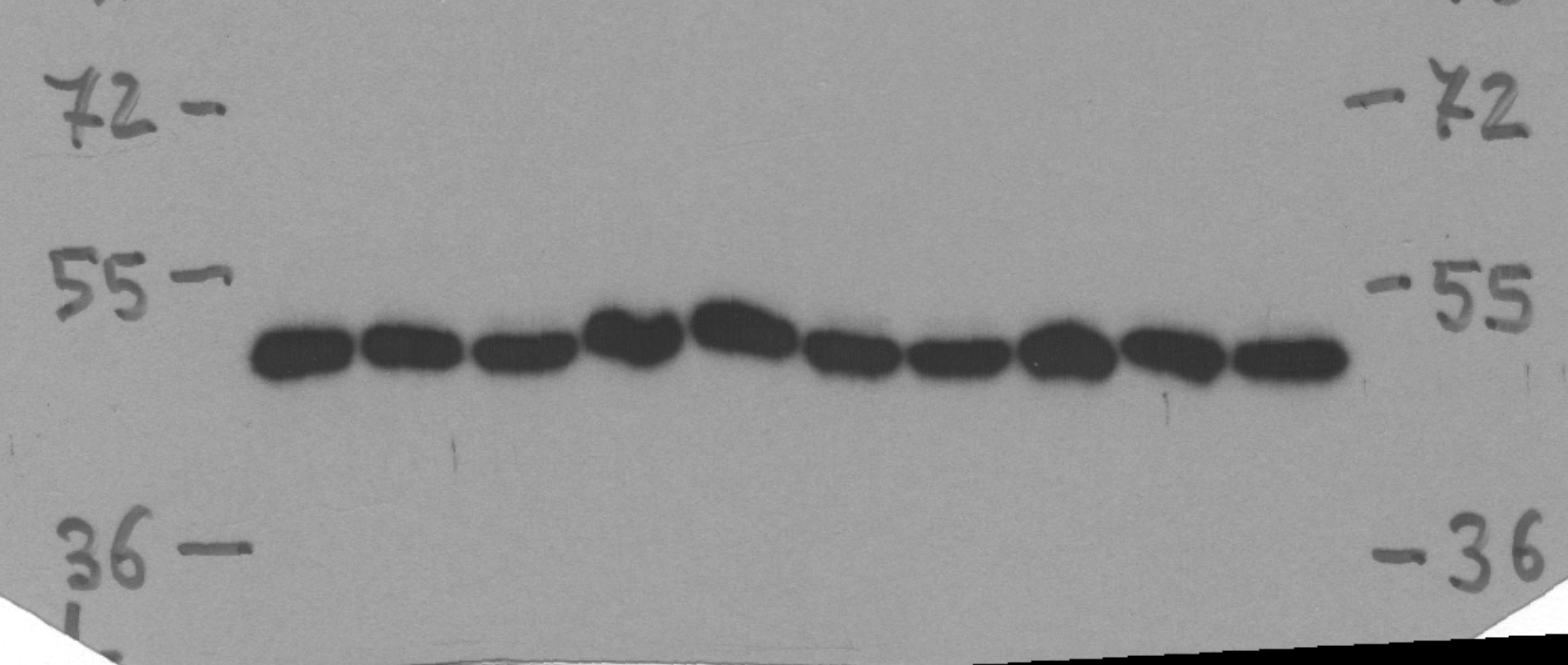


**Figure 2D**

Anti-Pan TrkB


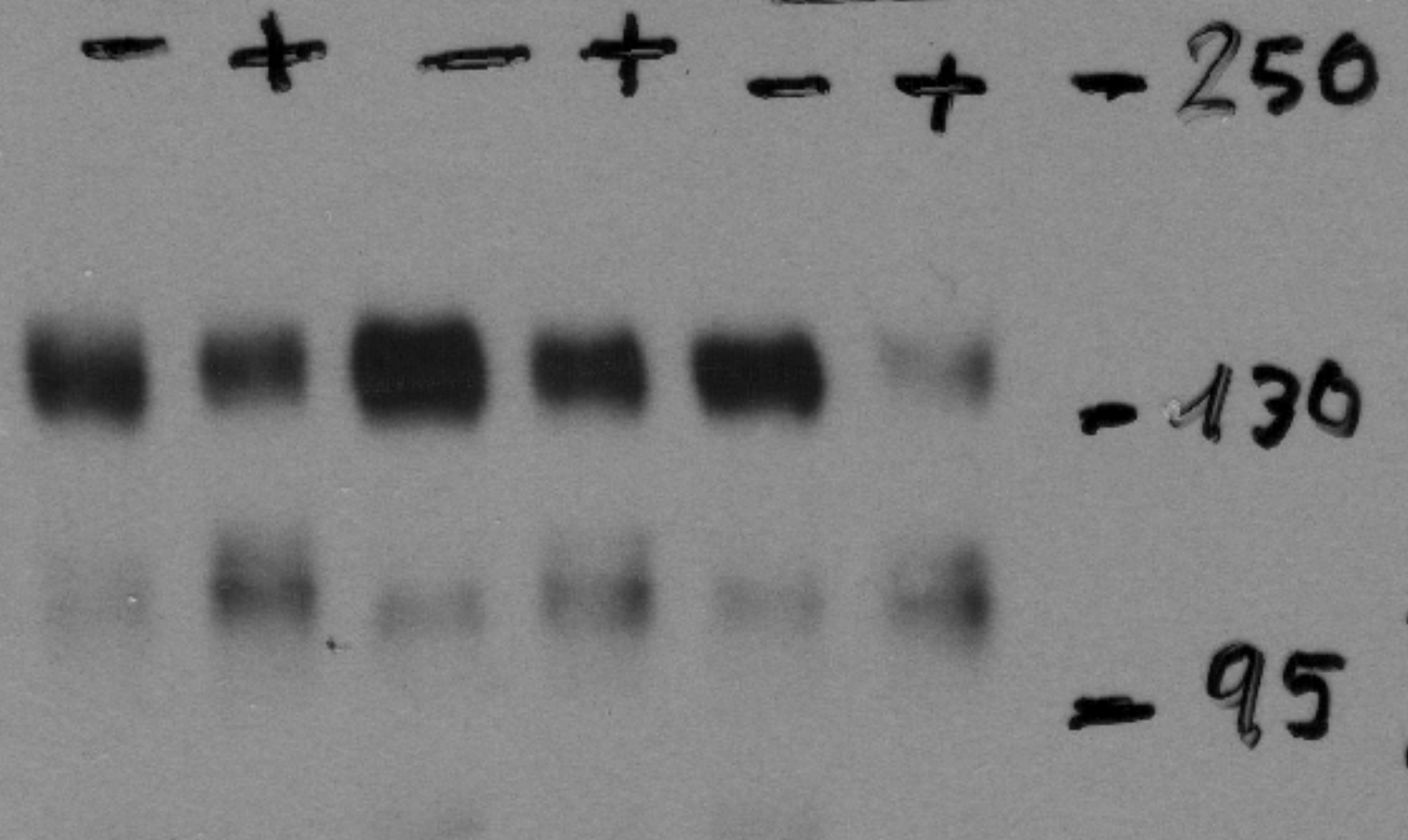


Anti-Spectrin


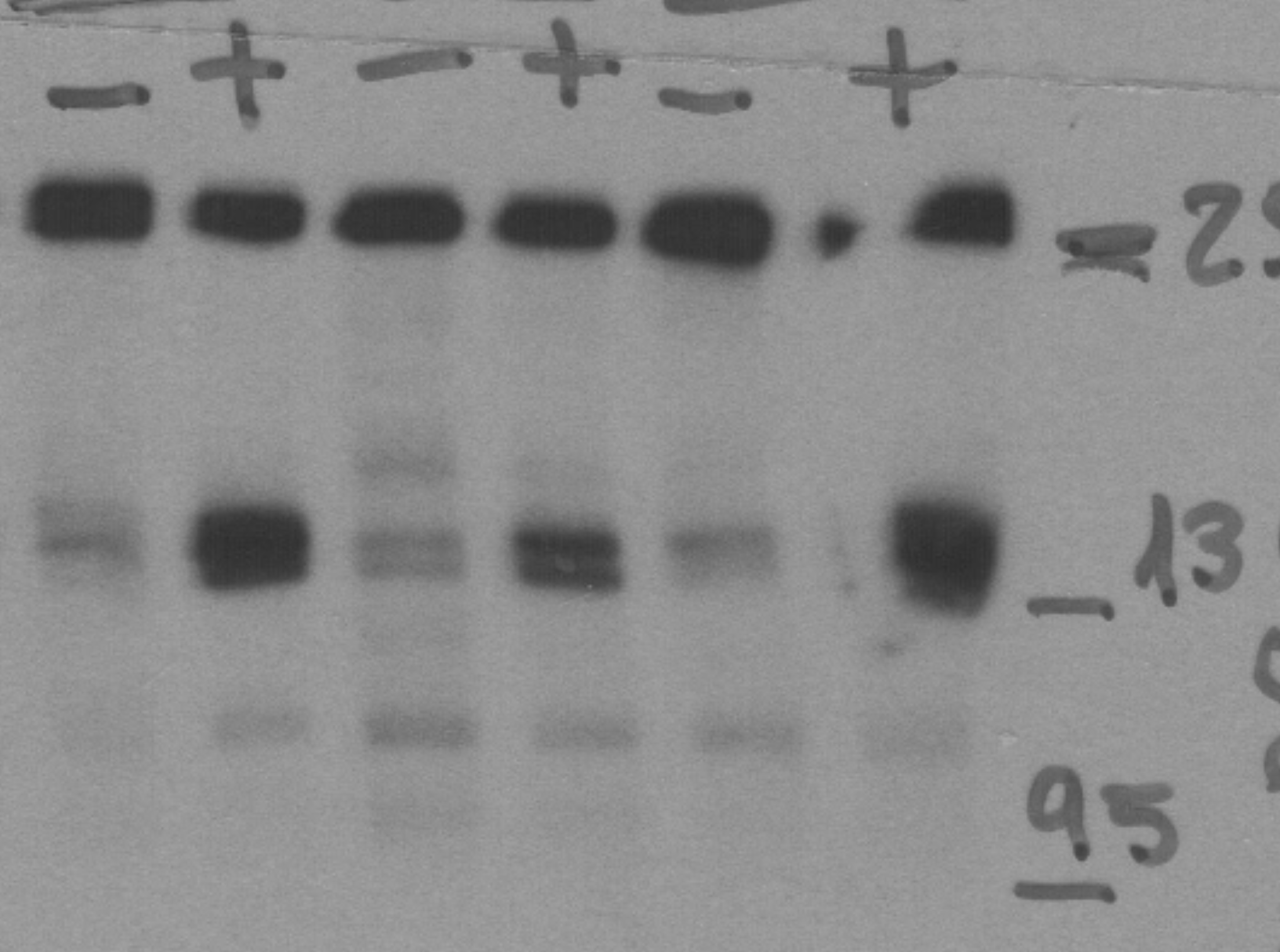


Anti-NSE


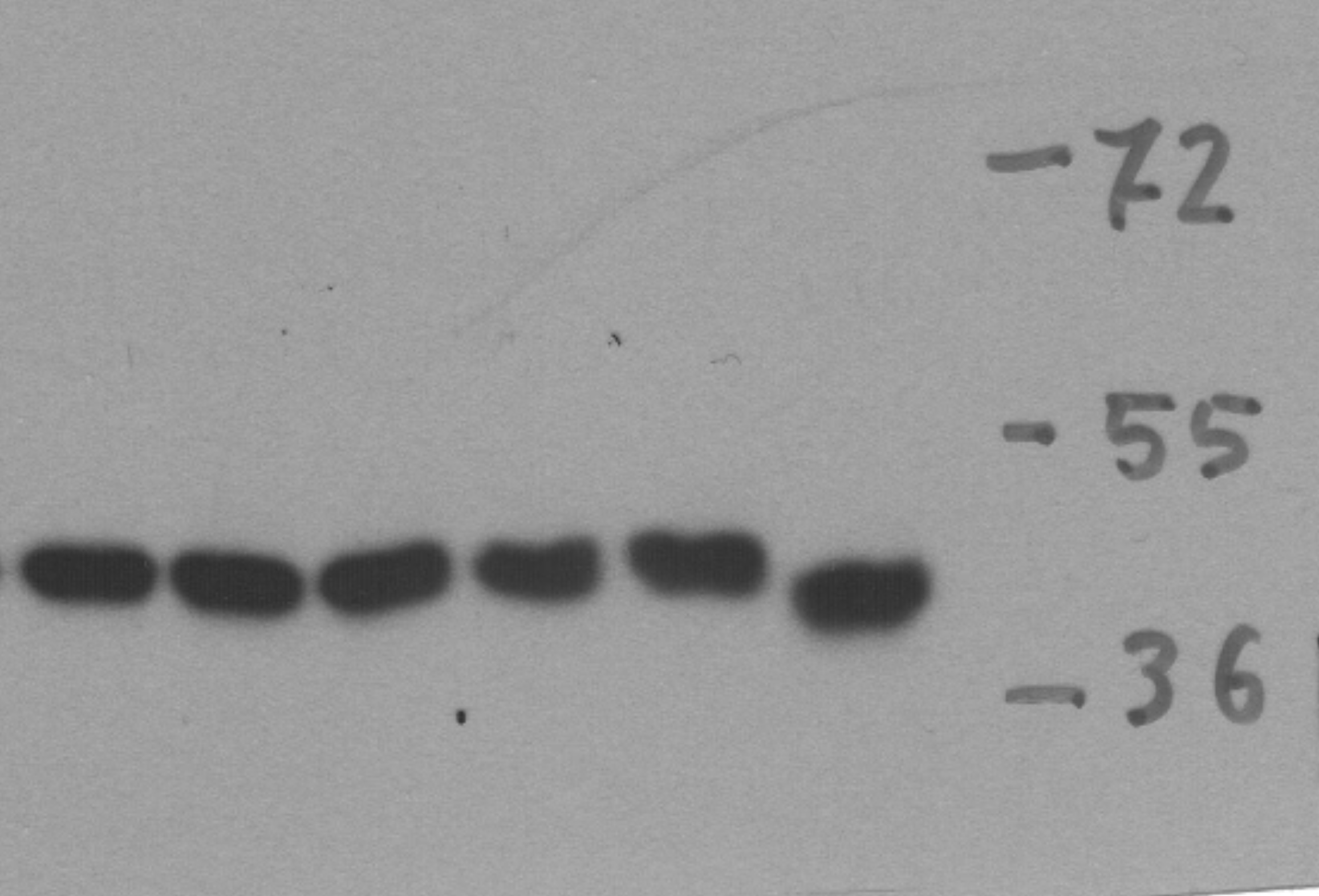

Supplement: Supplementary file 5 — Source Data for Figure 2 [file EMMM-11-e9950-s003.docx]
