# Supplementary figures and images for: Prevention of excitotoxicity‐induced processing of BDNF receptor TrkB‐FL leads to stroke neuroprotection
Source: EMBO Mol Med. 2019 Jun 3;11(7):e9950. doi: 10.15252/emmm.201809950 (PMC6609917; doi:10.15252/emmm.201809950)

**Source data Figure 3**

**Figure 3B**

Anti-Pan TrkB


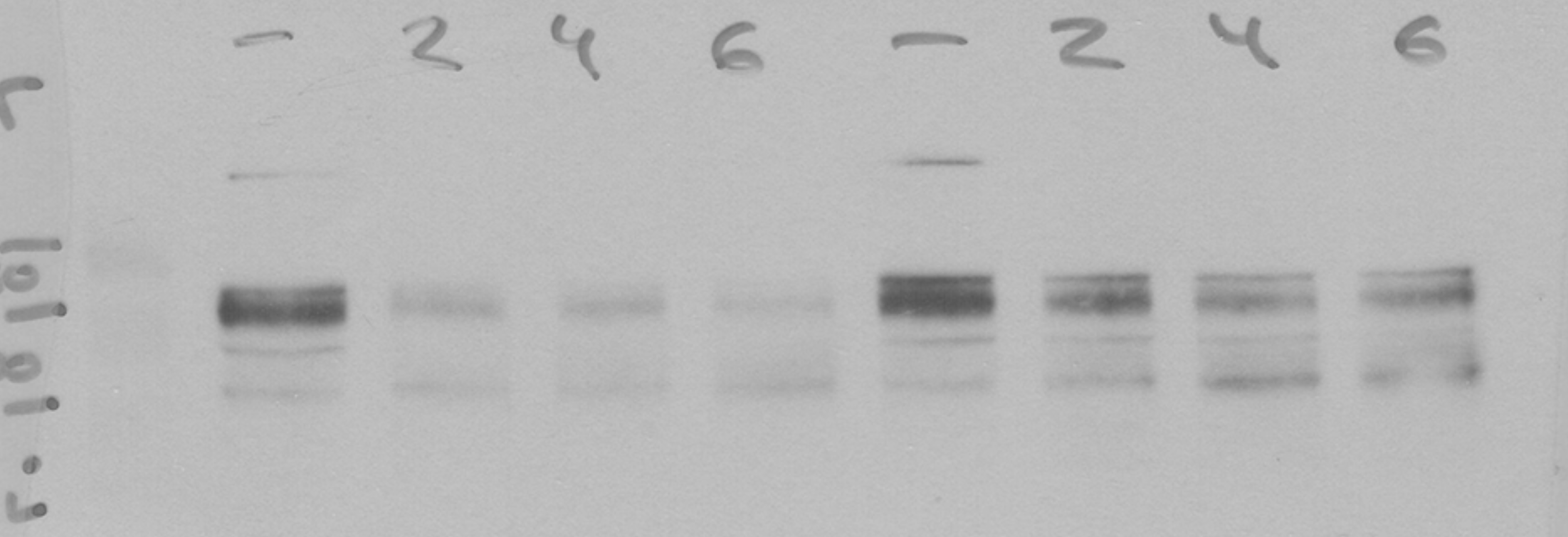


Anti-pY515 TrkB


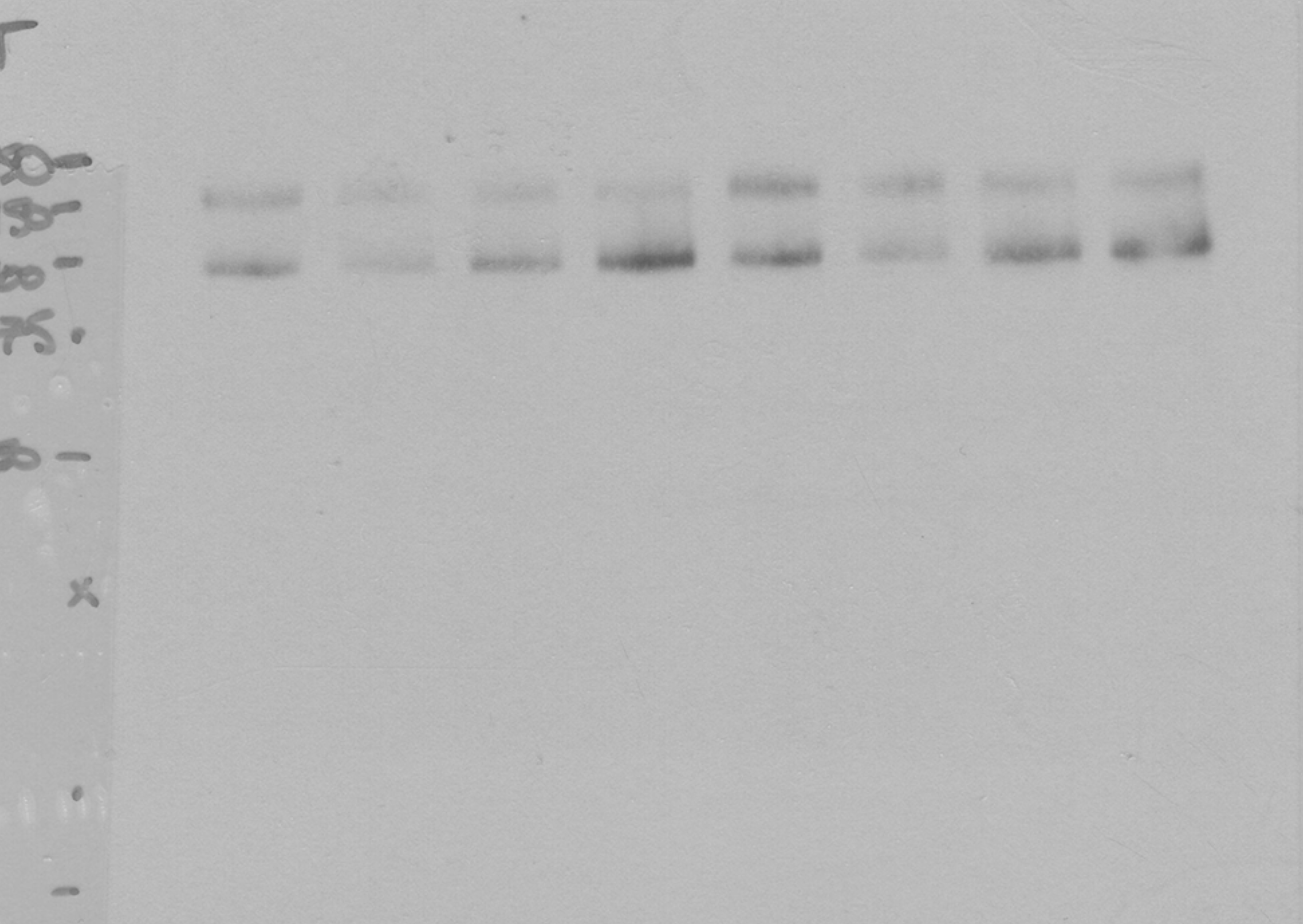


Anti-pY816 TrkB


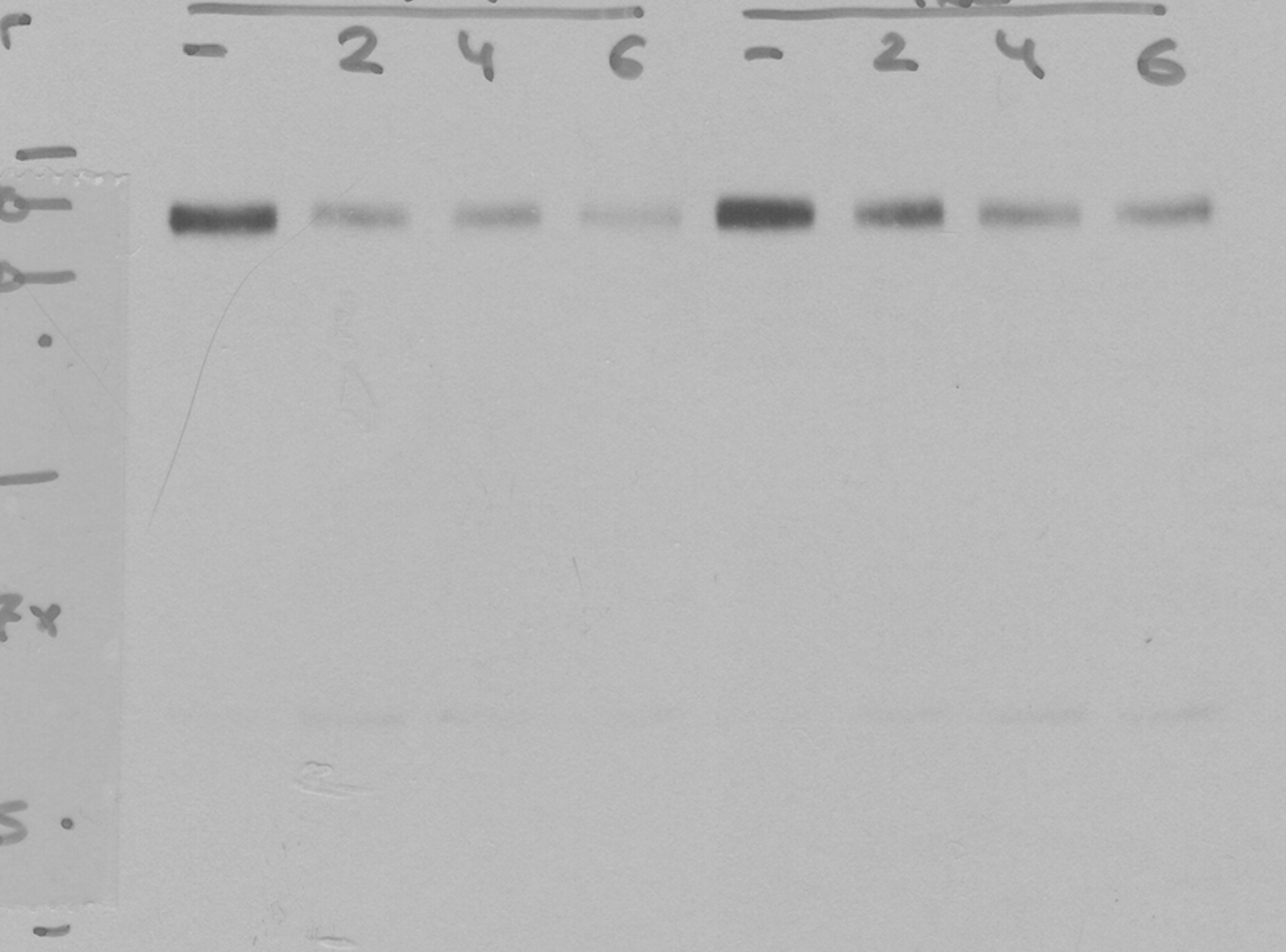


Anti-NSE


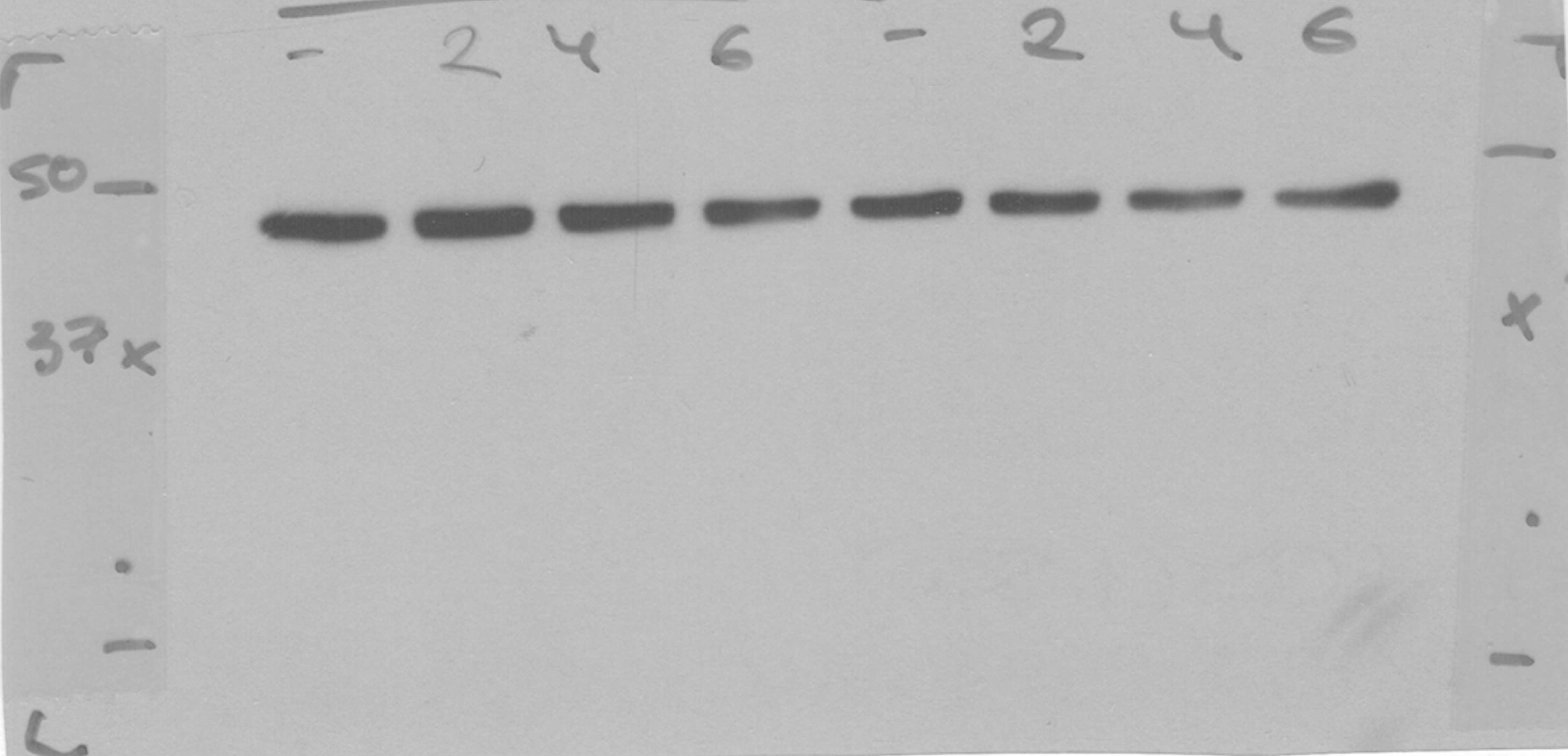


**Figure 3D**

Anti-pY816 TrkB

**
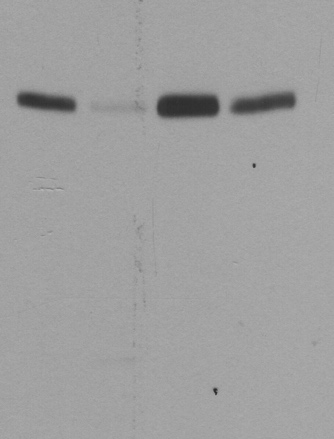

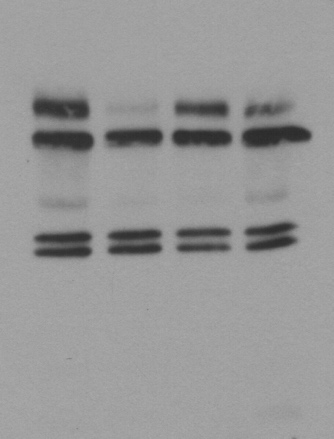
**

Supplement: Supplementary file 6 — Source Data for Figure 3 [file EMMM-11-e9950-s004.docx]

**Source data Figure 7**

**Figure 7C**

TrkB-FL Ct

**
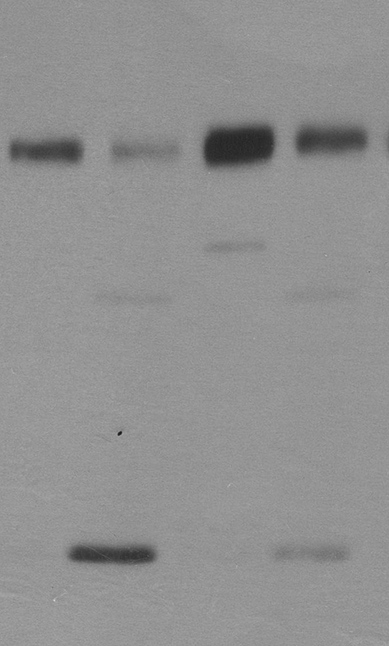
**

Anti-Spectrin


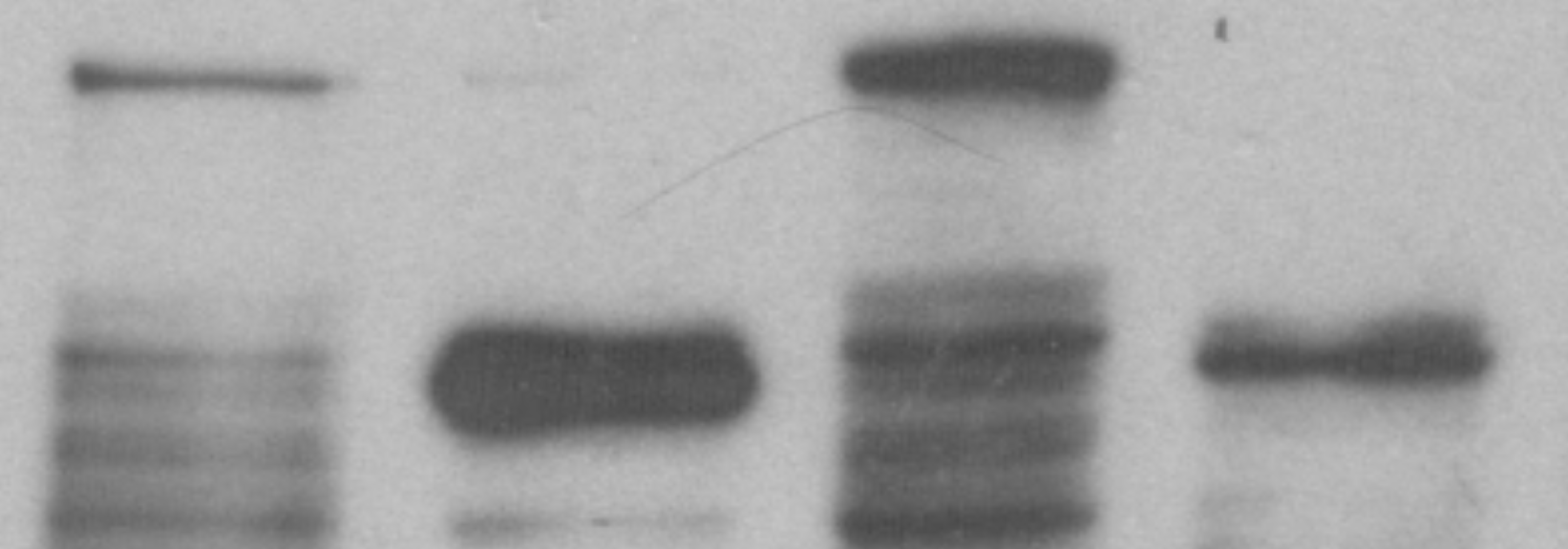


Anti-NSE


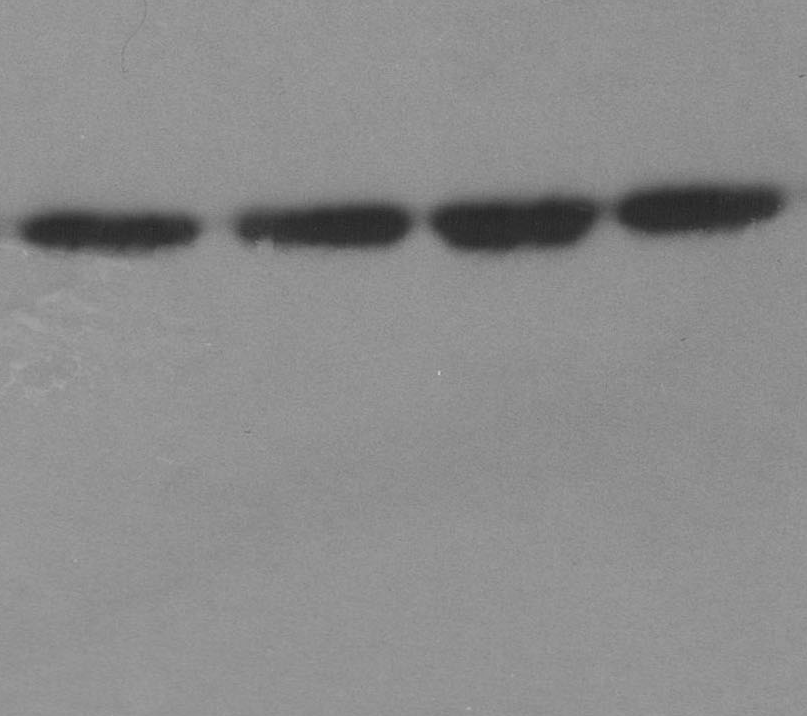

Supplement: Supplementary file 9 — Source Data for Figure 7 [file EMMM-11-e9950-s007.docx]
