# Supplementary material for: Prevention of excitotoxicity‐induced processing of BDNF receptor TrkB‐FL leads to stroke neuroprotection
Source: EMBO Mol Med. 2019 Jun 3;11(7):e9950. doi: 10.15252/emmm.201809950 (PMC6609917; doi:10.15252/emmm.201809950)

**Source data Figure 4**

**Figure 4A**

Anti-Pan TrkB


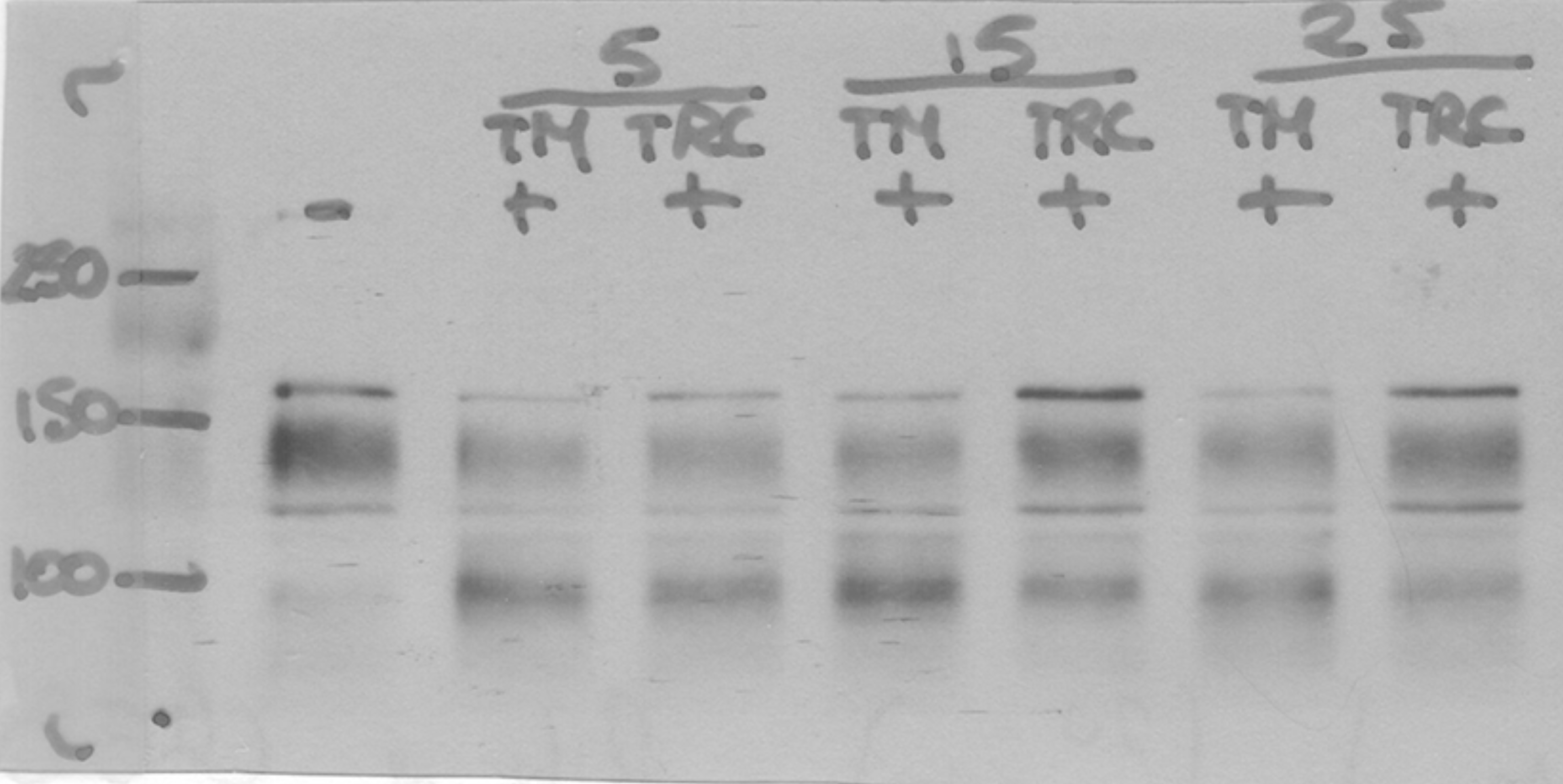


Anti-CREB


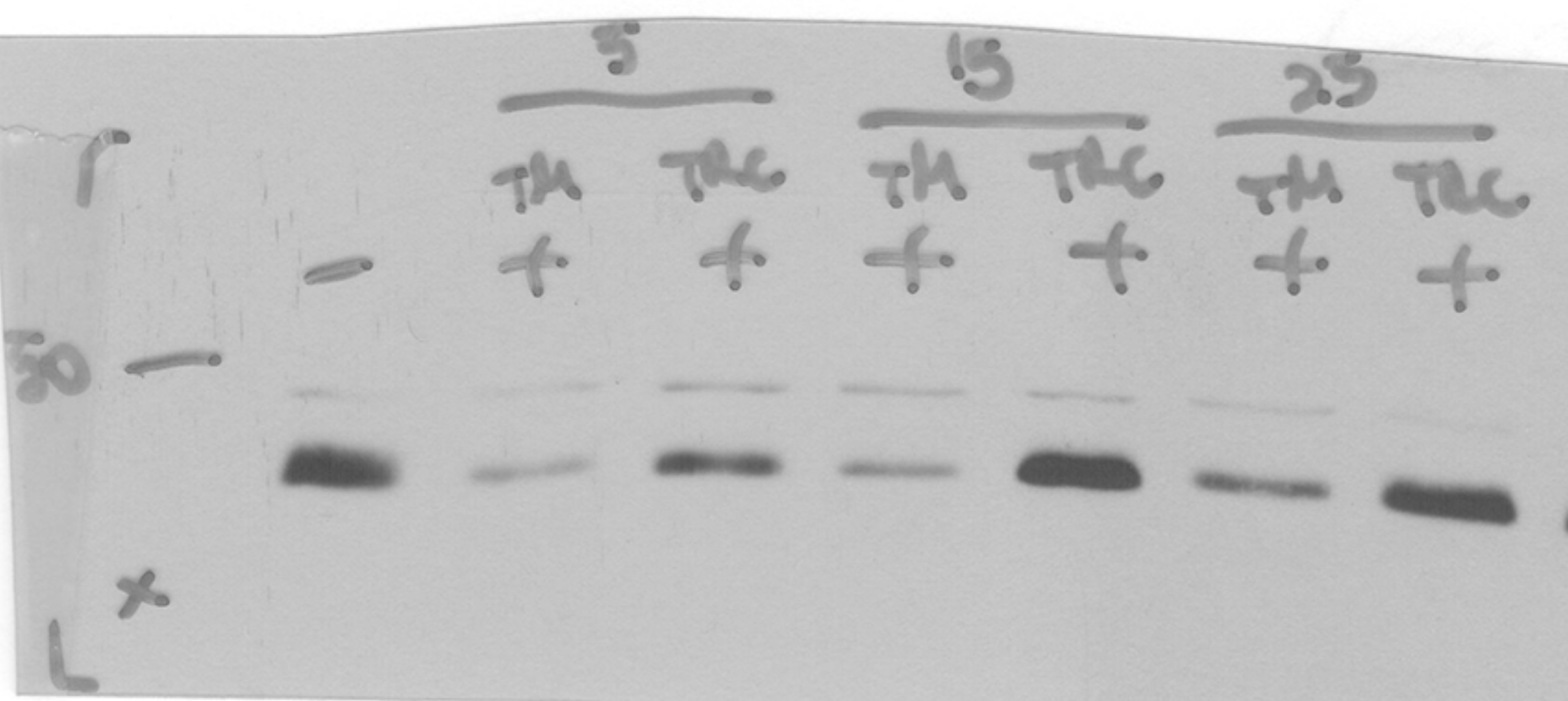


Anti-pCREB


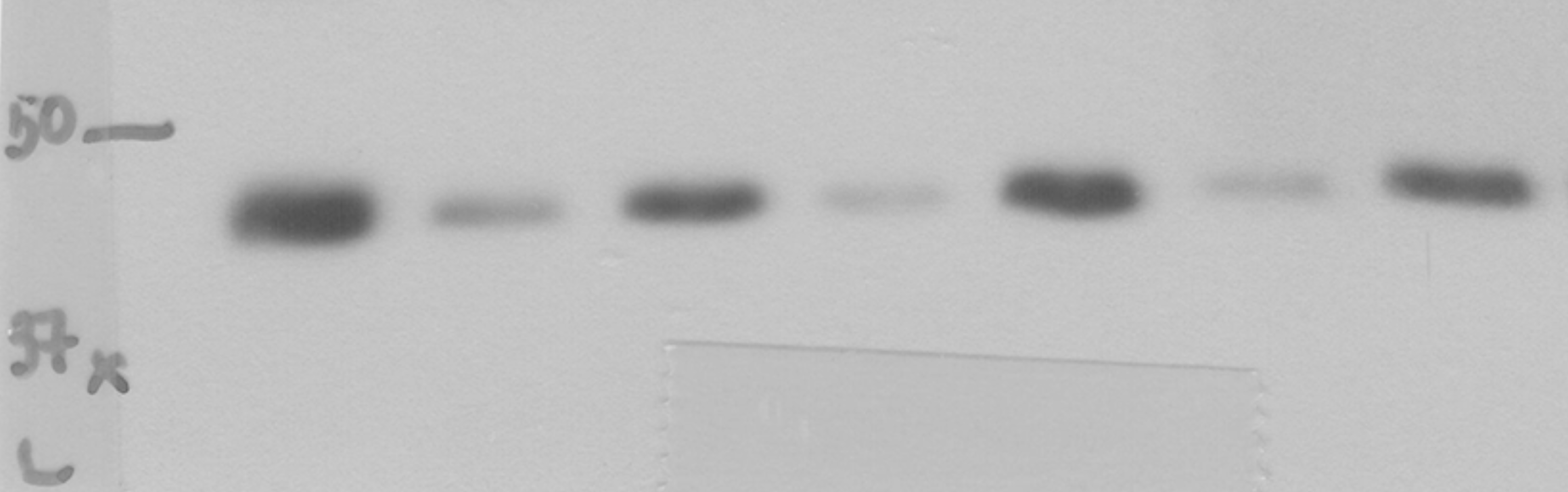


Anti-Spectrin


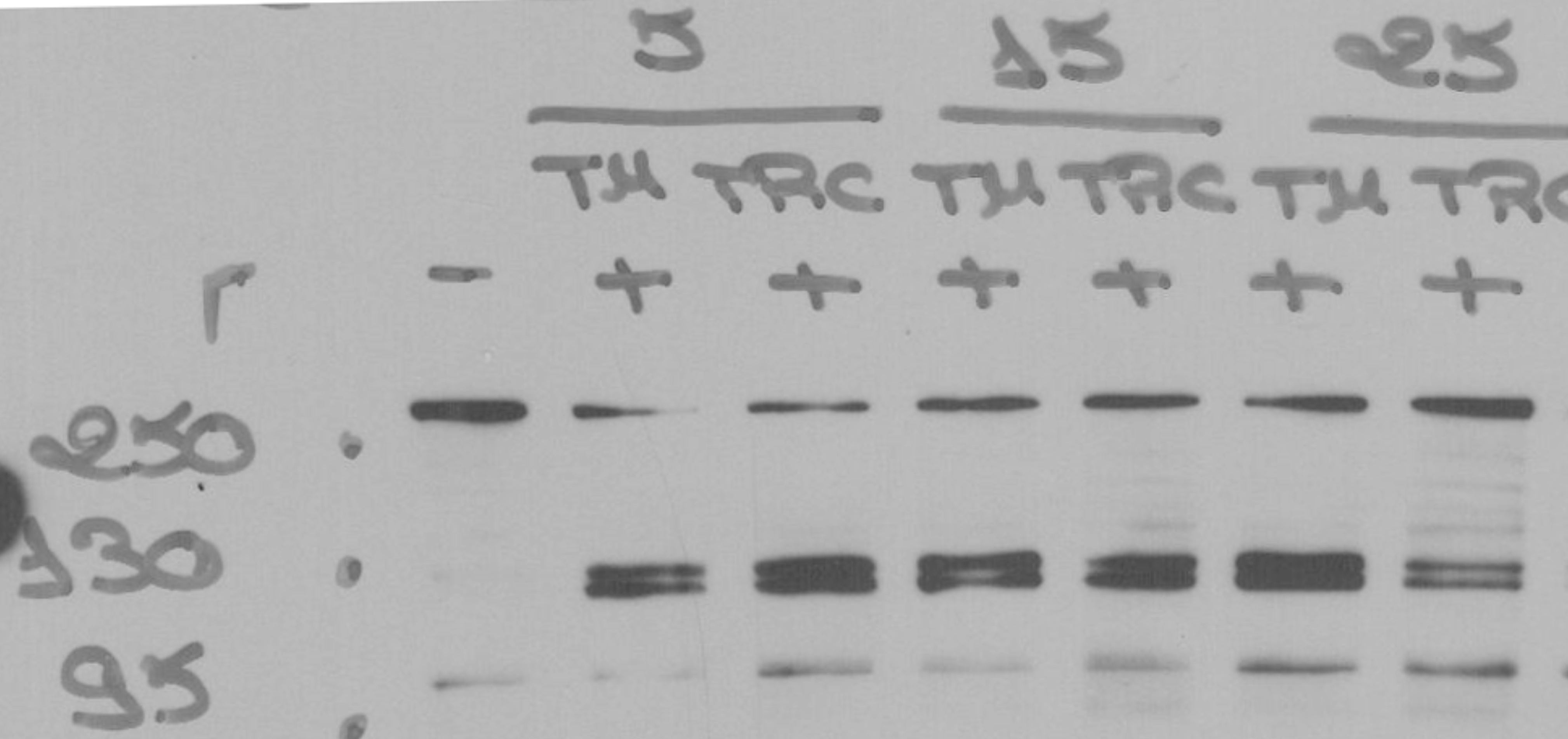


Anti-NSE


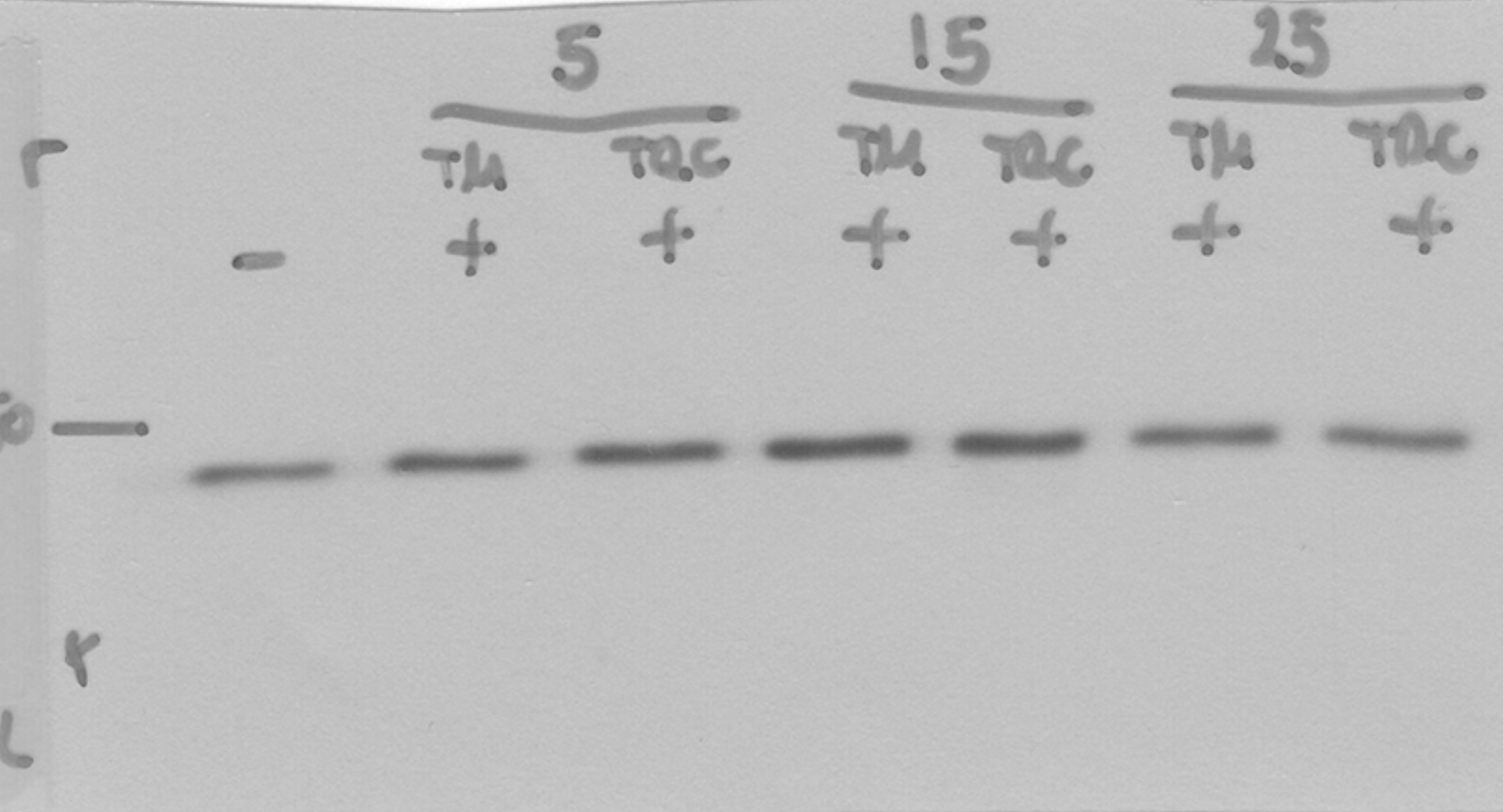


**Figure 4B**

Anti-Pan TrkB


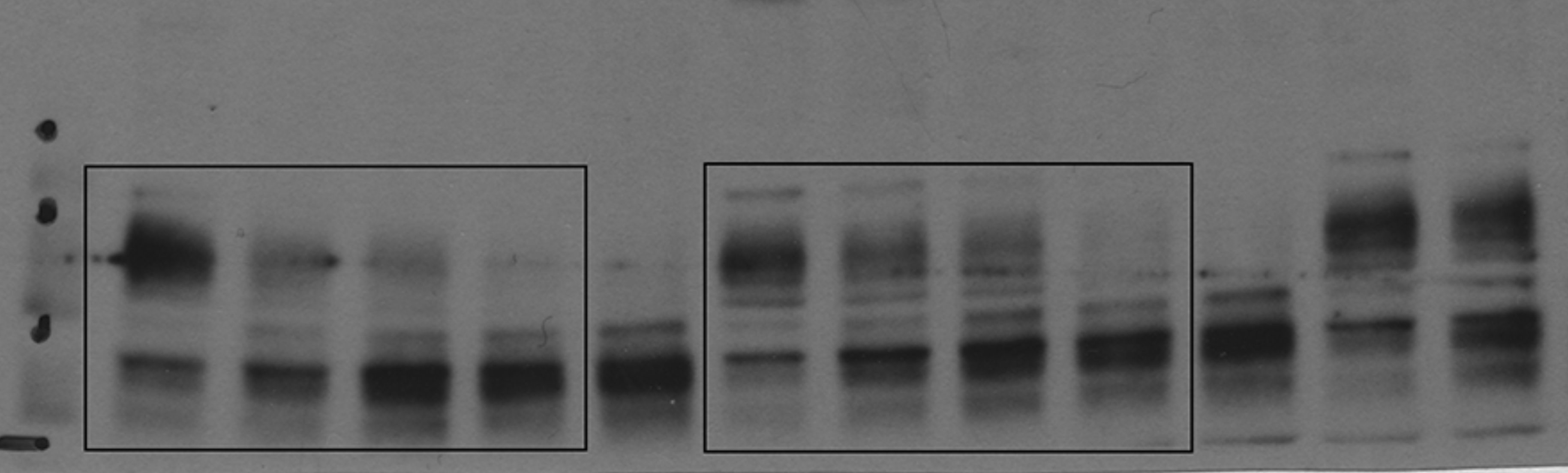


Anti-TrkB-T1


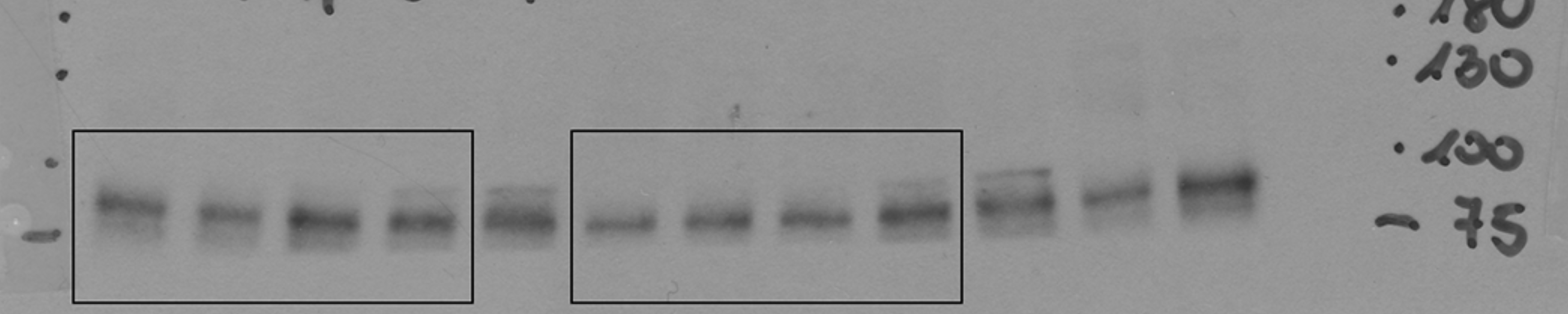


Anti-CREB


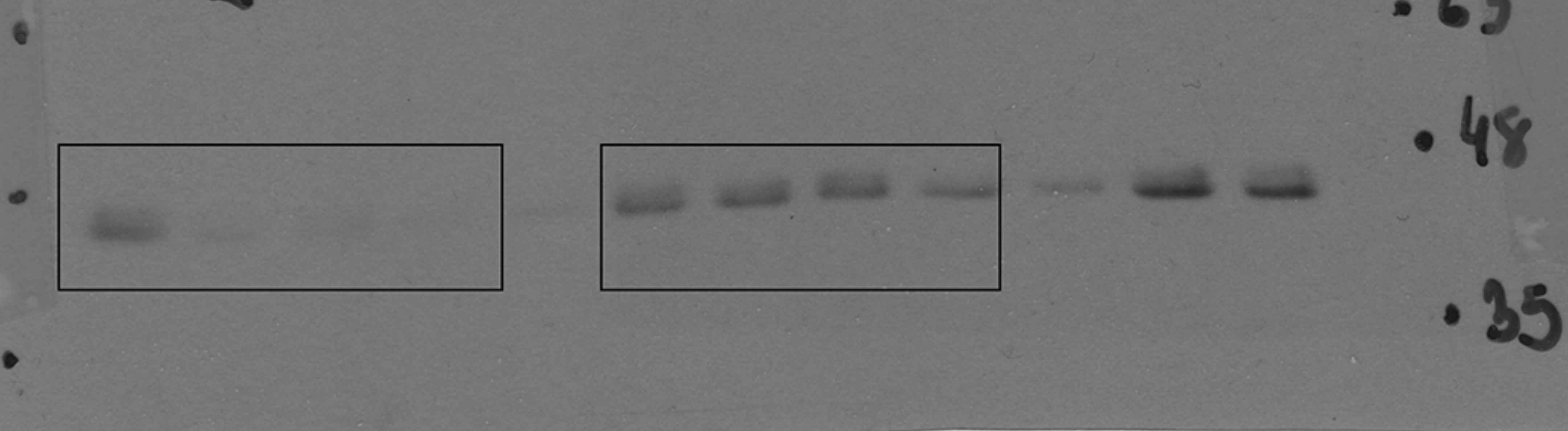


Anti-pCREB


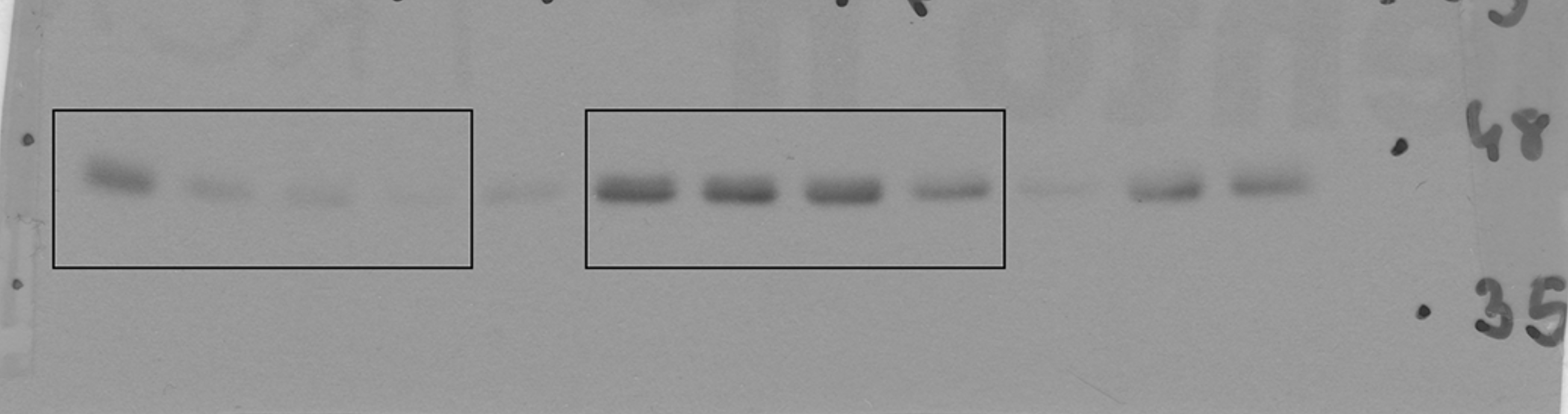


Anti-MEF2D


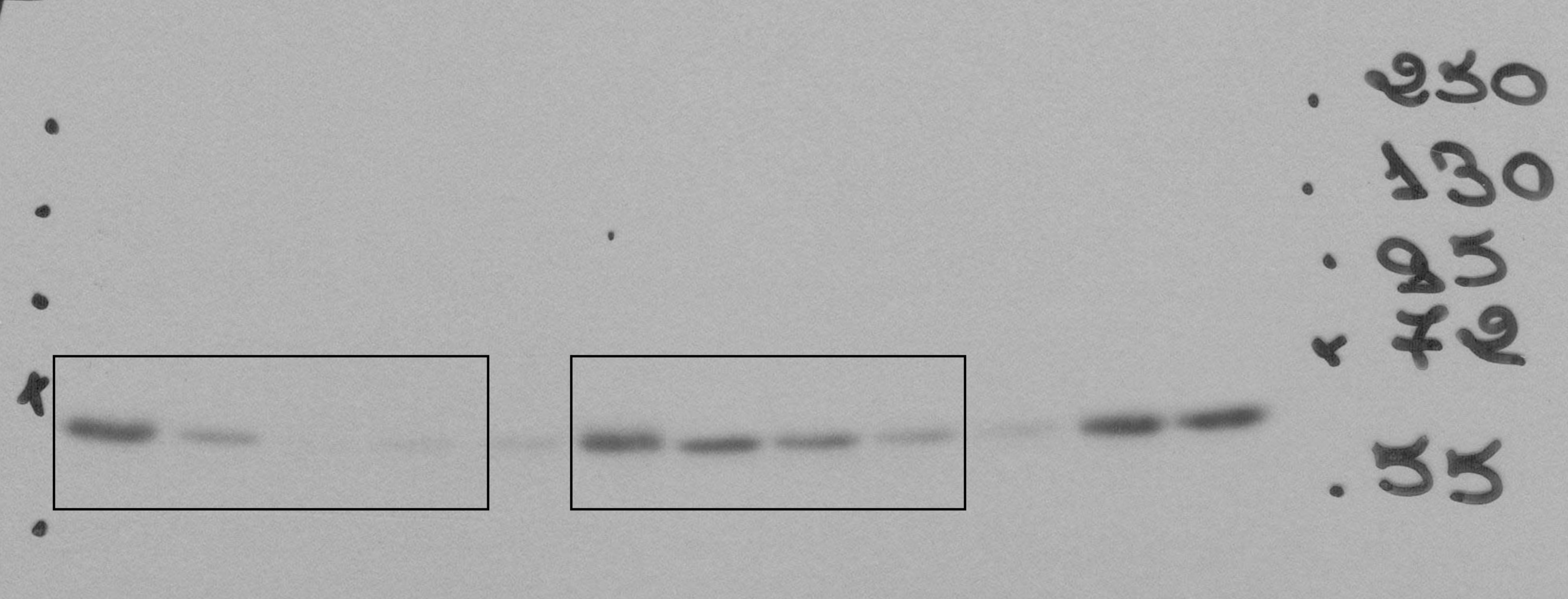


Anti-GluN1


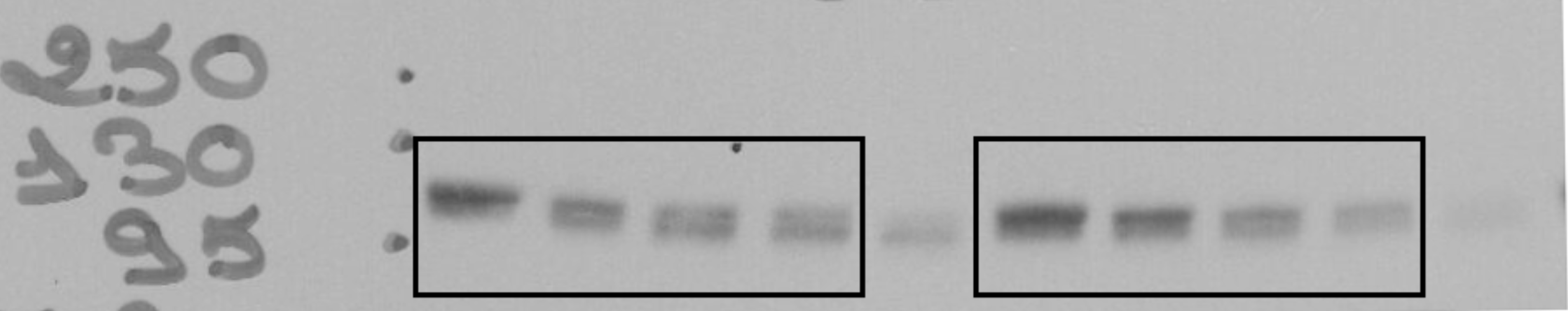


Anti-GluN2A


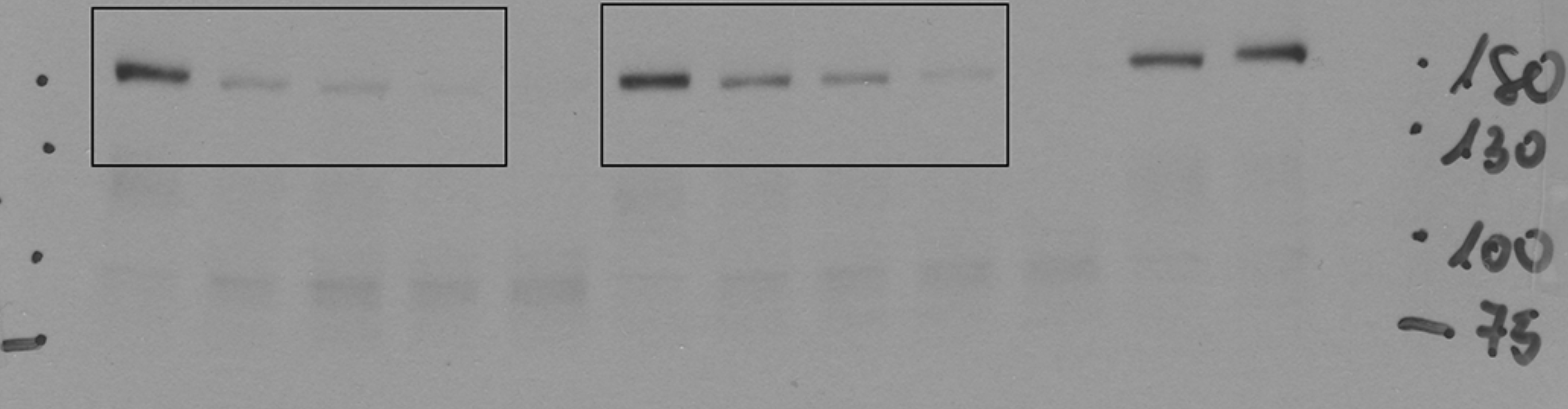


Anti-Spectrin


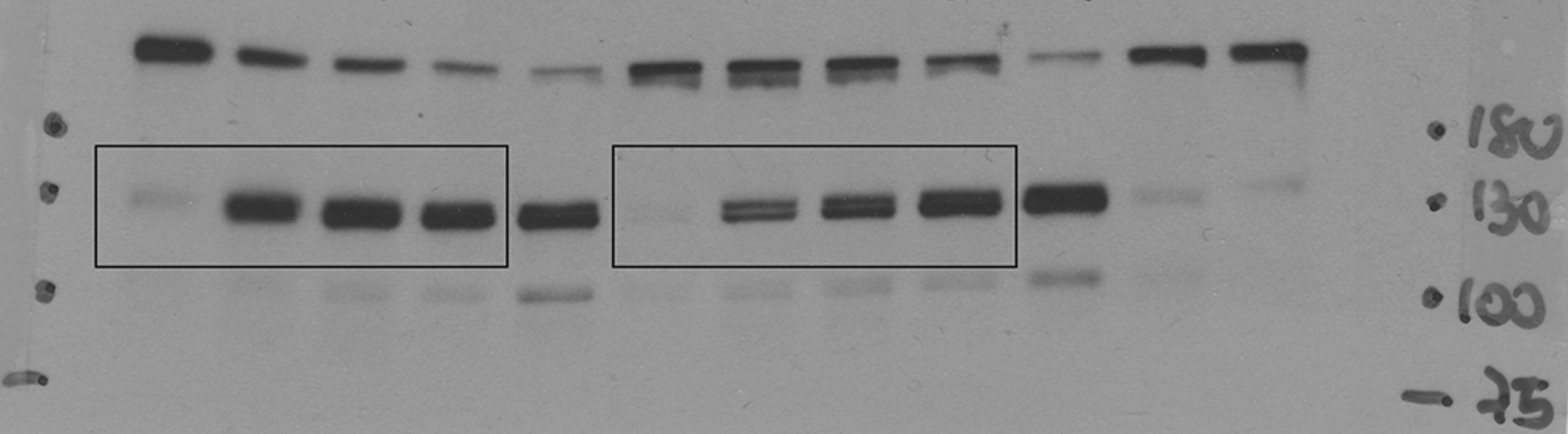


Anti-NSE


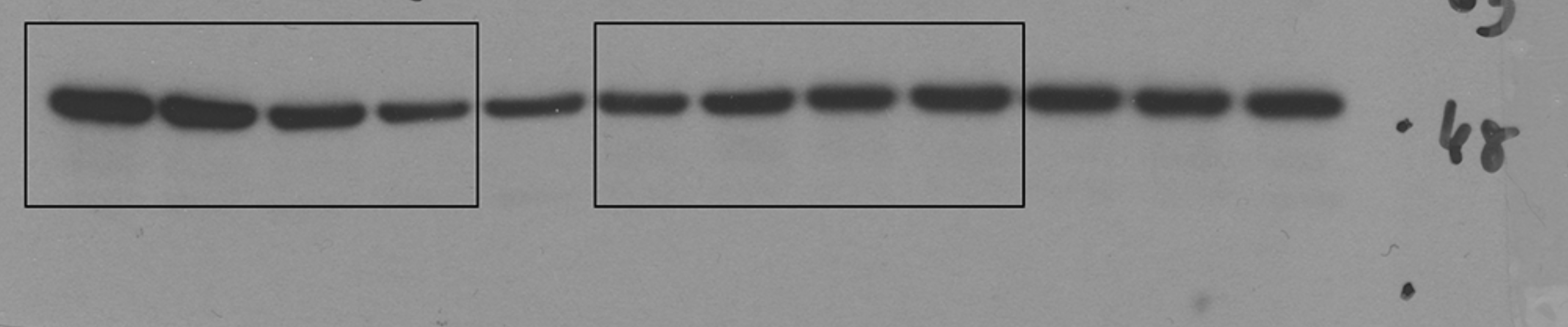

Supplement: Supplementary file 7 — Source Data for Figure 4 [file EMMM-11-e9950-s005.docx]
