# Supplementary material for: Prevention of excitotoxicity‐induced processing of BDNF receptor TrkB‐FL leads to stroke neuroprotection
Source: EMBO Mol Med. 2019 Jun 3;11(7):e9950. doi: 10.15252/emmm.201809950 (PMC6609917; doi:10.15252/emmm.201809950)

**Source data Figure 6**

**Figure 6A**

TrkB-FL Ct (three different exposures)

**
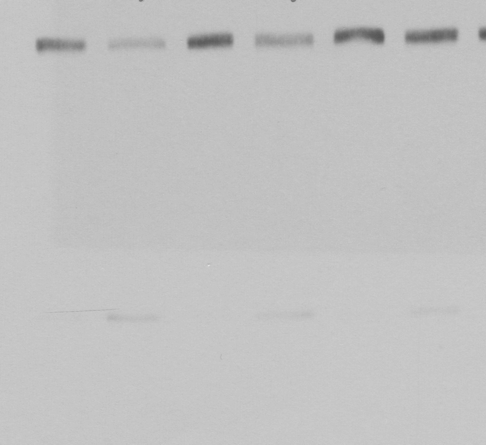
**

**
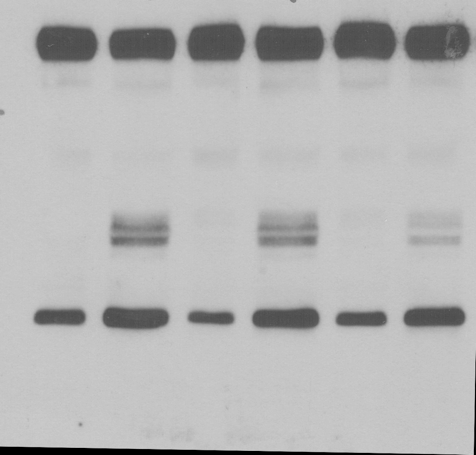
**

**
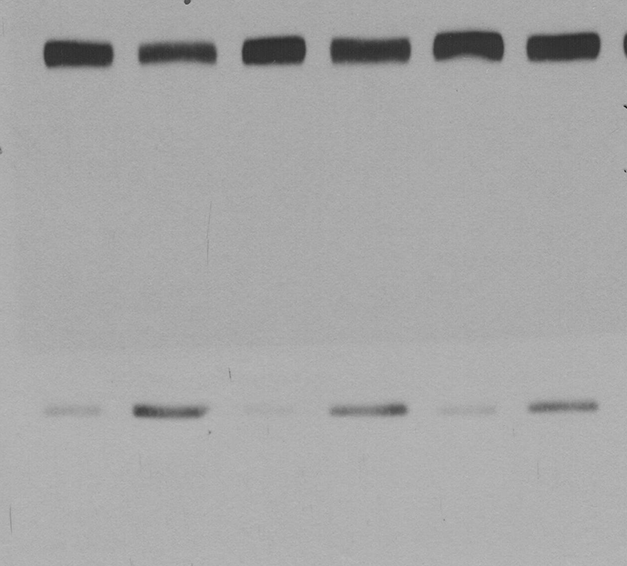
**

Anti-NSE

**
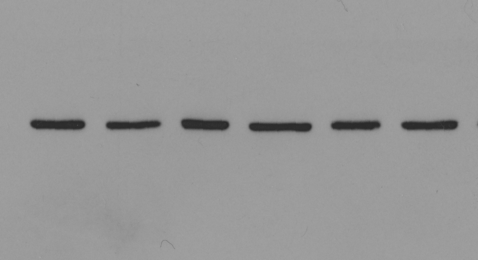
**

**Figure 6E**

Total TrkB-FL


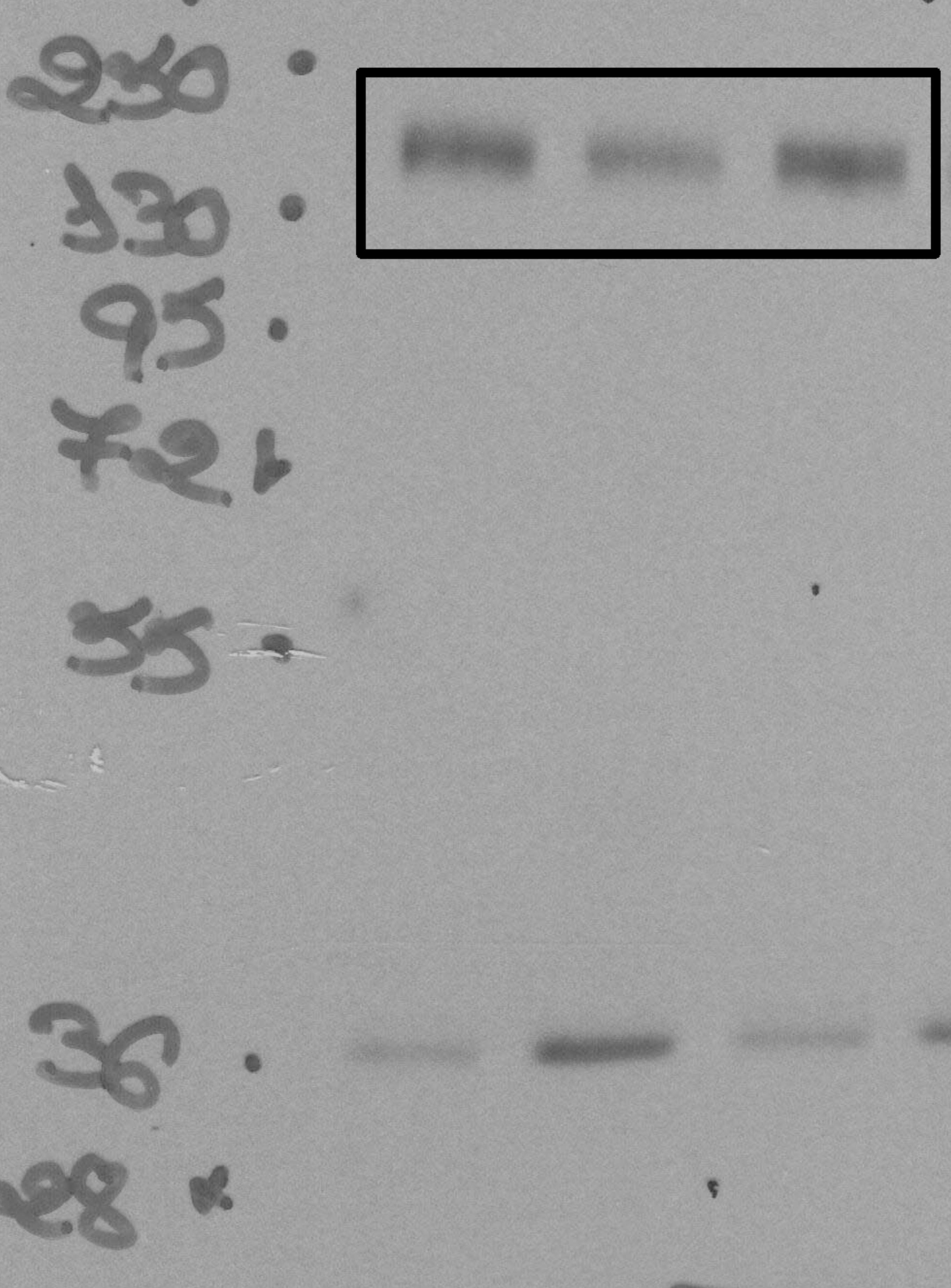


Surface TrkB-FL


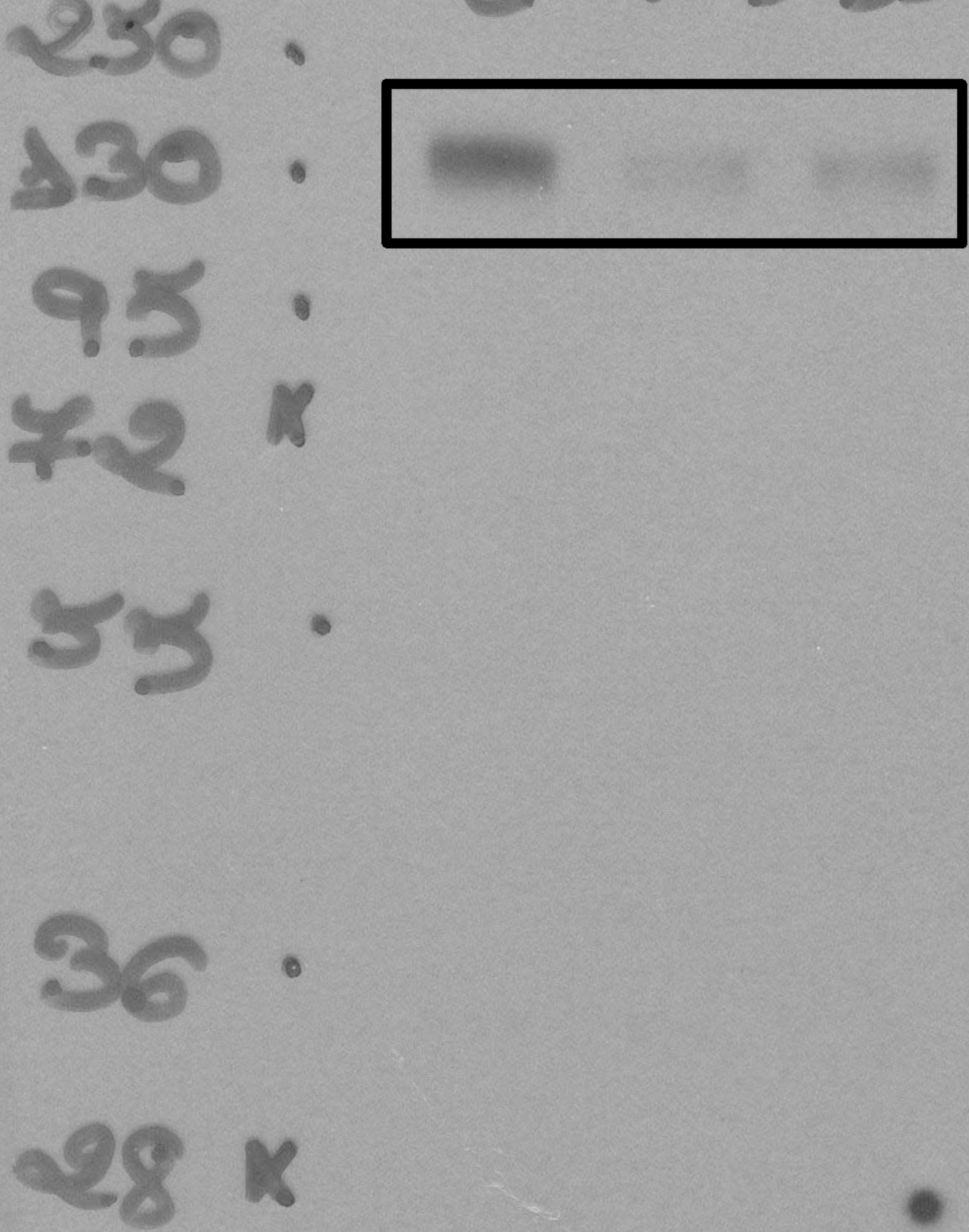


**Figure 6F**

Total TrkB-FL


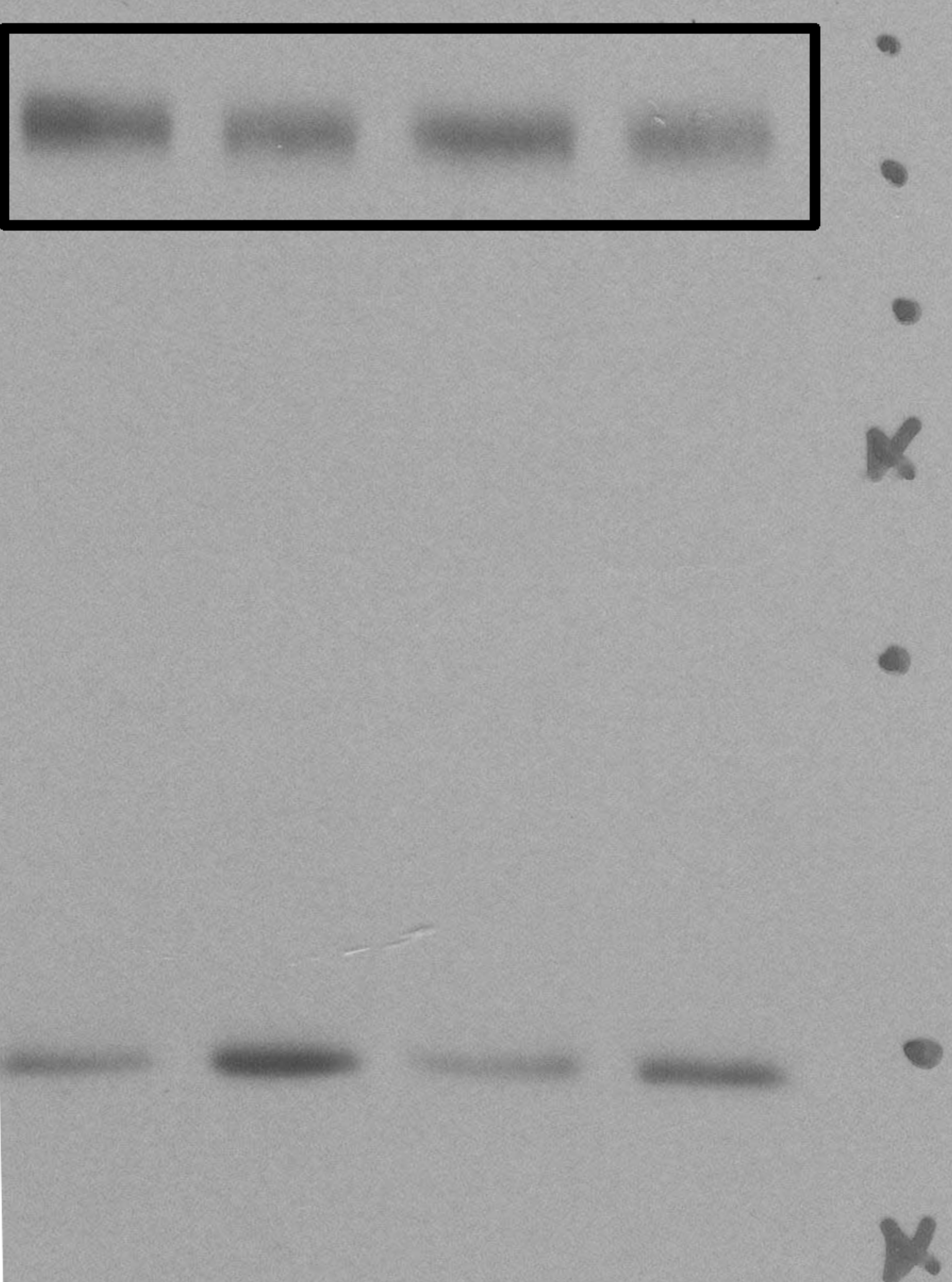


Surface TrkB-FL


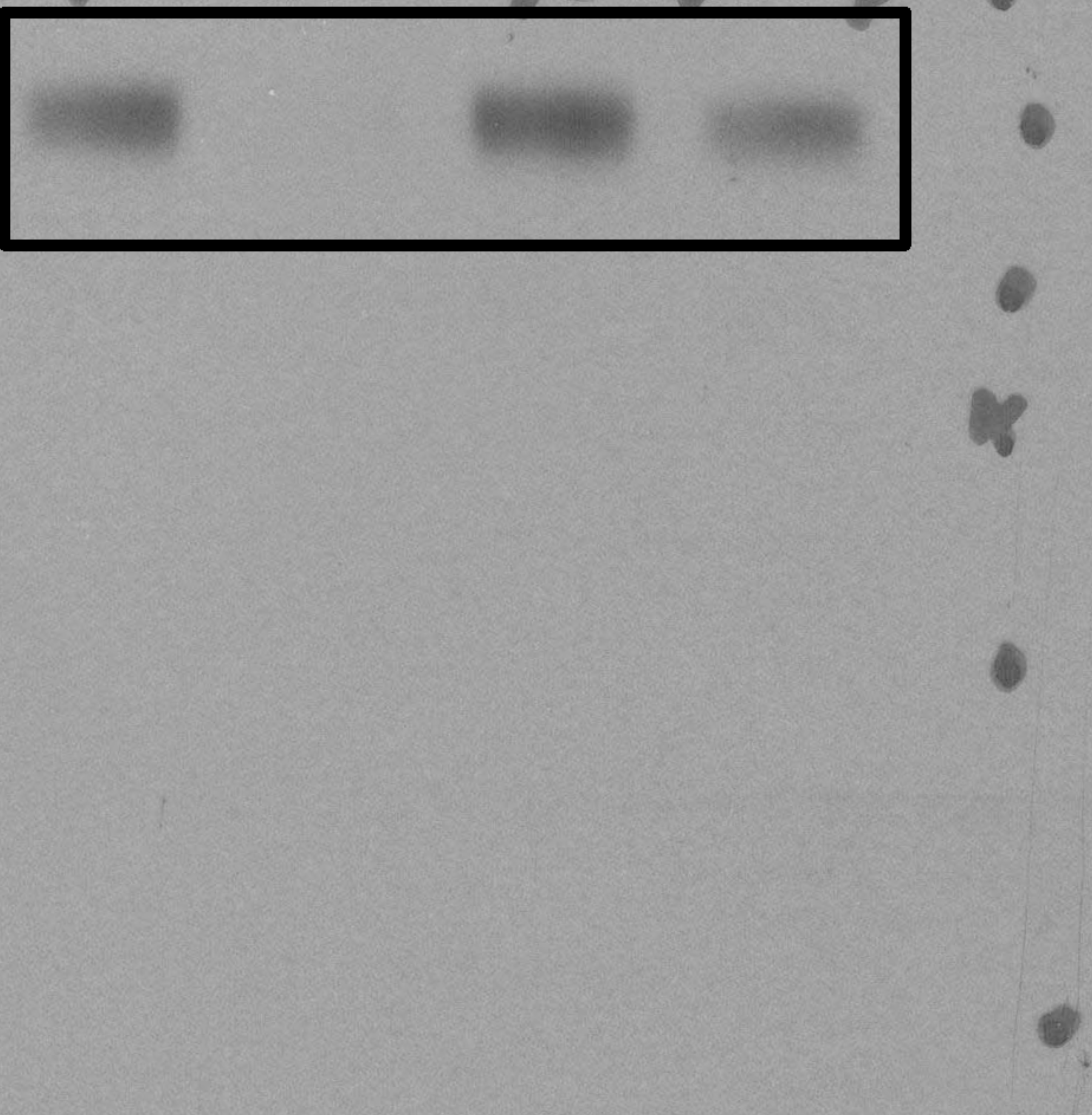


**Figure 6H**

TrkB-FL Ct


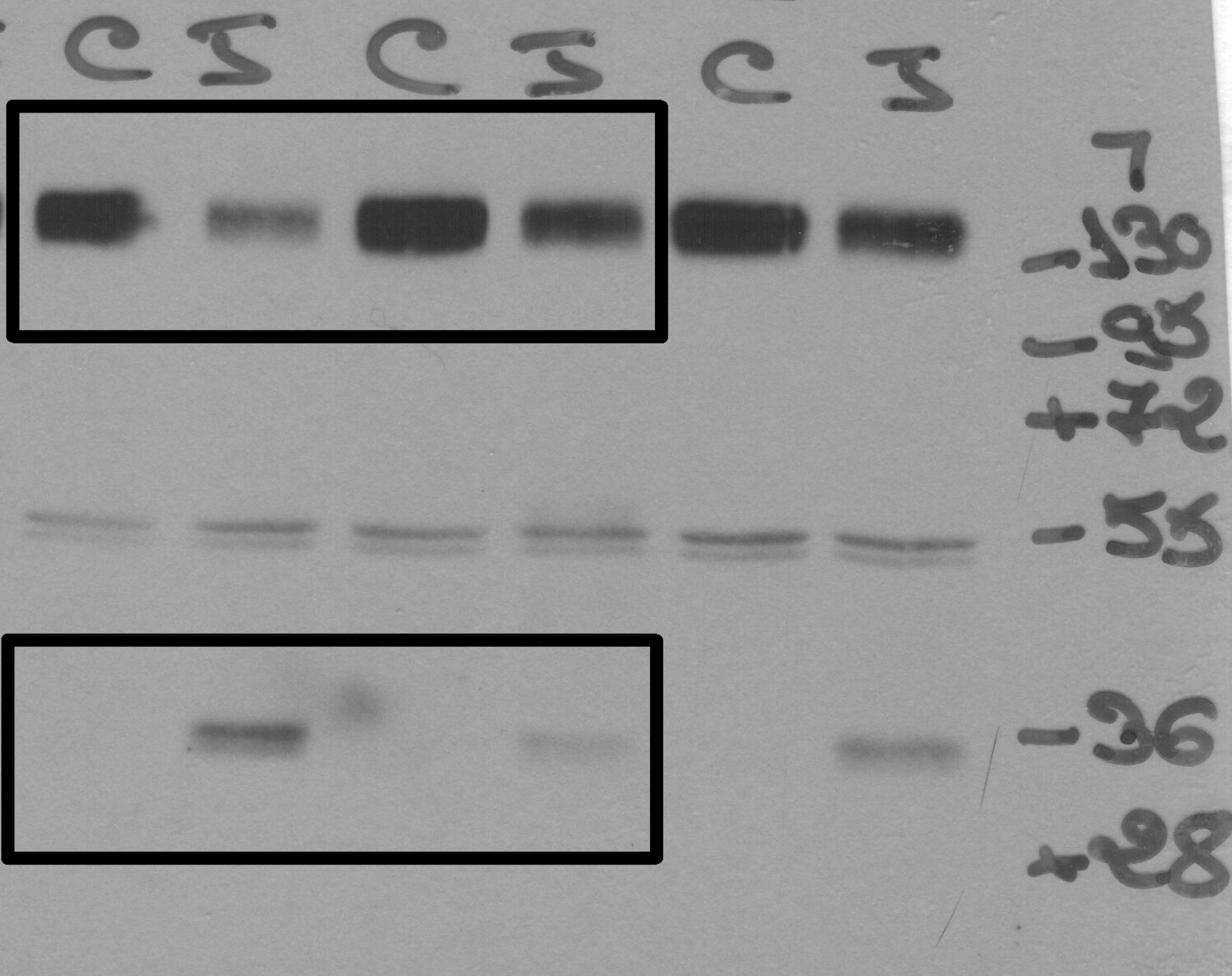


Anti-TrkB-T1


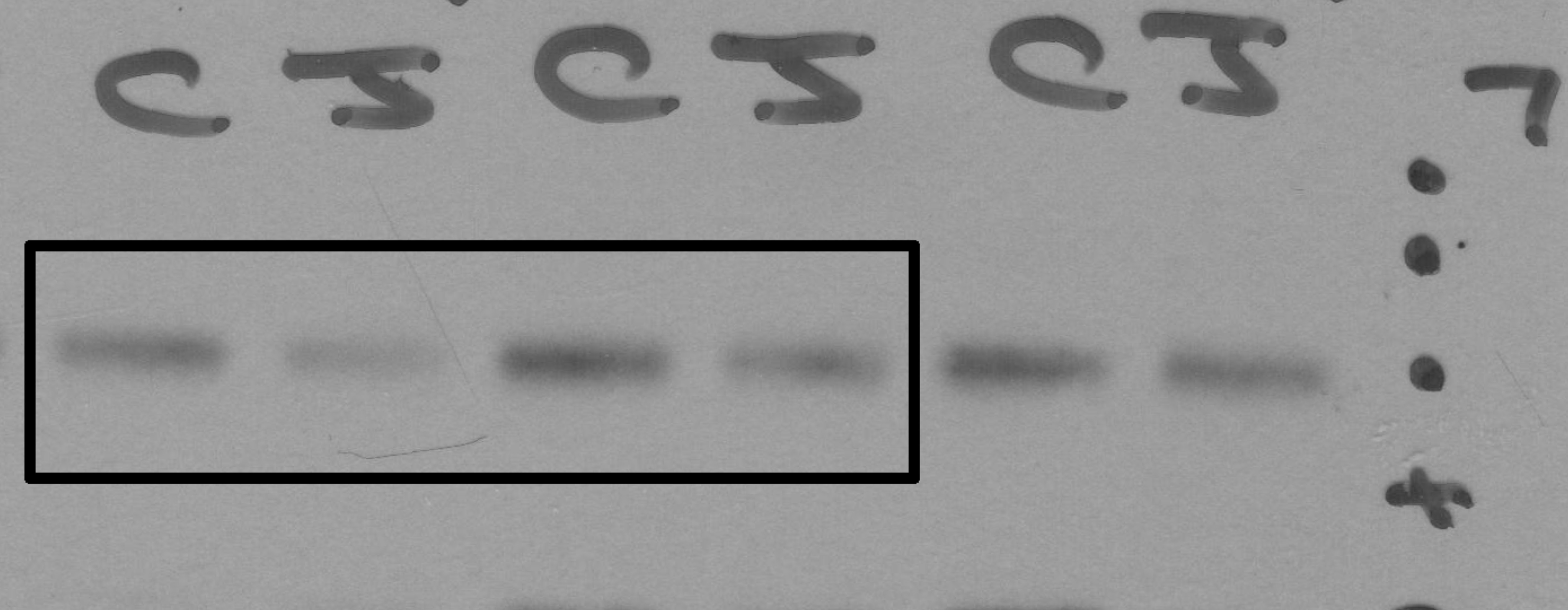


Anti-Spectrin


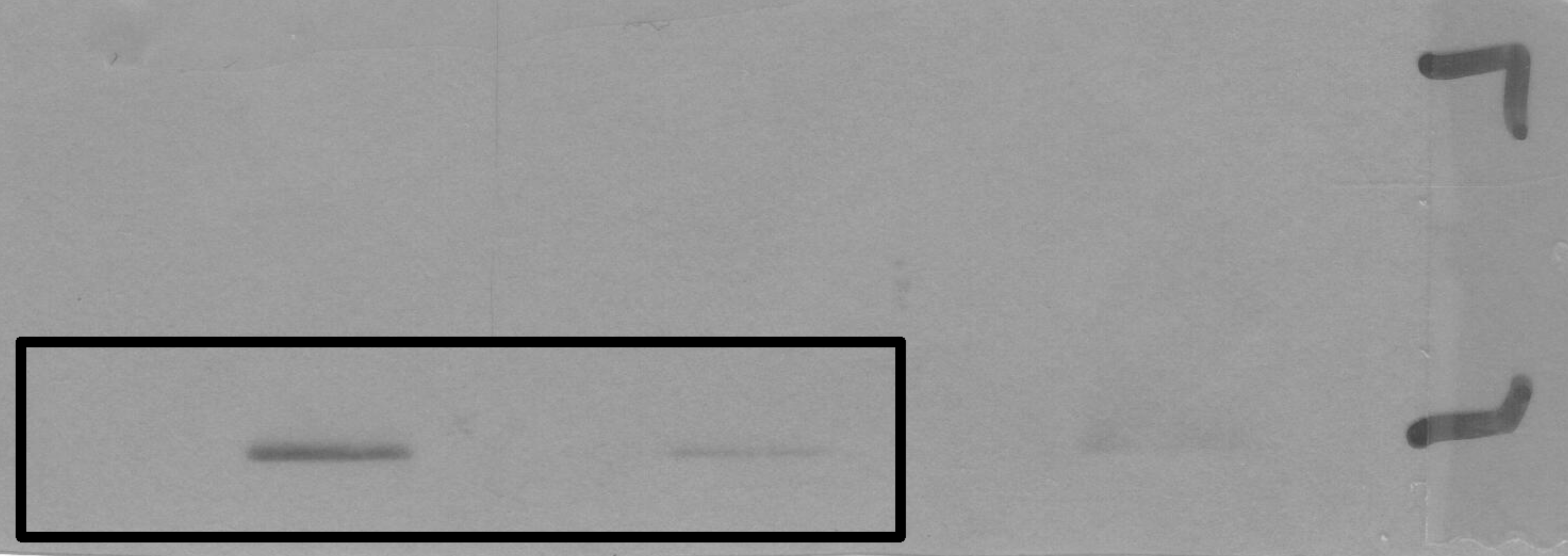


Anti-NSE


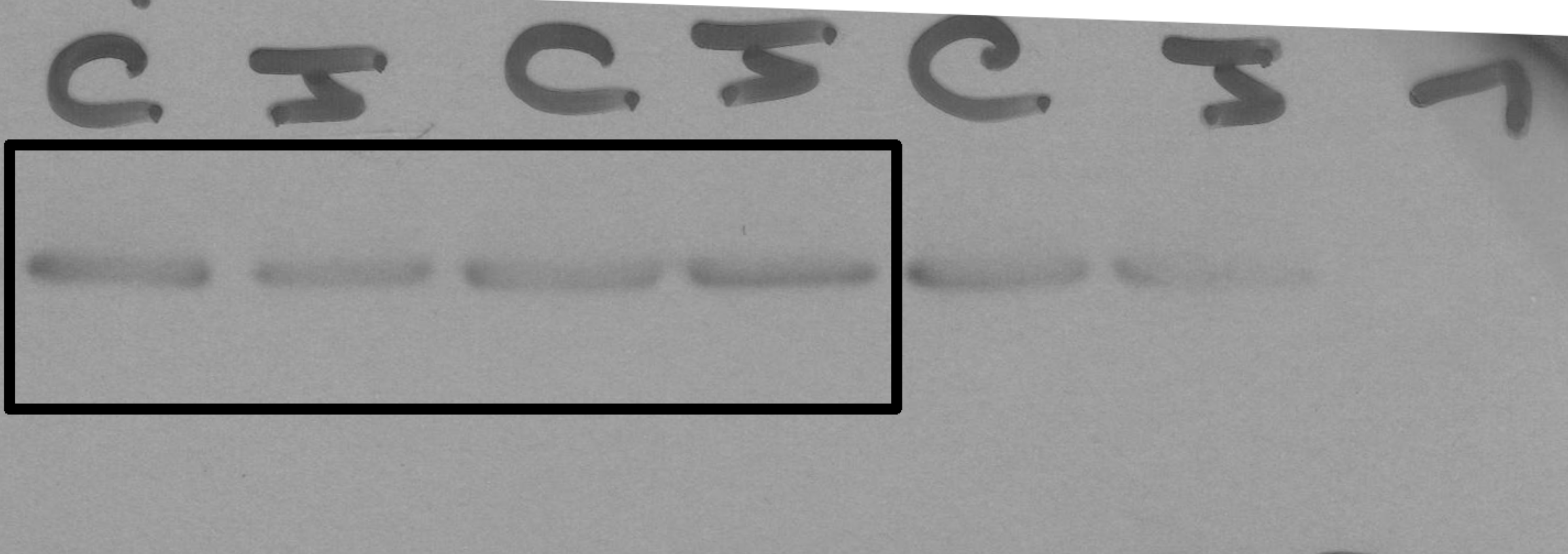

Supplement: Supplementary file 8 — Source Data for Figure 6 [file EMMM-11-e9950-s006.docx]
